# Hesperetin Attenuates Experimentally Induced Skeletal Muscle Dysfunction by Modulating Time-of-Day-Dependent Gene Expression and Mitochondrial Redox-Related Markers

**DOI:** 10.3390/antiox15091185

**Published:** 2026-09-17

**Authors:** Suhyeon Lee, Yumin Sim, Minkyeong Lee, Eunju Kim

**Affiliations:** Department of Food and Nutrition, Kongju National University, 54 Daehak-ro, Yesan 32439, Chungcheongnam-do, Republic of Korea

**Keywords:** circadian rhythm, skeletal muscle aging, hesperetin, mitochondrial function, oxidative stress

## Abstract

Skeletal muscle aging is characterized by impaired myogenic differentiation, mitochondrial dysfunction, oxidative stress, and circadian rhythm disruption, contributing to sarcopenia and muscle atrophy. Hesperetin, a natural flavonoid, has antioxidant and mitochondrial protective effects; however, its role in skeletal muscle circadian regulation during aging remains unclear. This study investigated the effects of hesperetin (20 µM or 100 mg/kg b.w.) using D-galactose (D-gal, 20 g/L)-induced senescent C2C12 myotubes, a D-gal (150 mg/kg b.w., i.p.)-induced aging mouse model, and a dexamethasone (Dex, 20 mg/kg b.w., i.p.)-induced muscle atrophy model. In D-gal-treated mice, hesperetin improved hanging test performance and increased SDH-positive area, particularly during the active phase. Hesperetin also partially modulated core clock gene expression and mitochondrial function-related gene expression. In D-gal-induced senescent C2C12 myotubes, hesperetin improved myotube formation, reduced SA-β-gal-positive cells, DCF-DA fluorescence, and MDA levels, enhanced antioxidant enzyme activities, and improved mitochondrial-associated functional indicators, including ATP levels, mitochondrial membrane potential-related fluorescence, *pMitoTimer*-based mitochondrial oxidation-associated signals, and *Ppargc1a* expression patterns. These findings suggest that hesperetin may protect against experimentally induced skeletal muscle dysfunction by modulating time-dependent gene expression, mitochondrial-related activities, and muscle-related functional markers.

## 1. Introduction

Skeletal muscle is one of the most dynamic and highly plastic tissues in the human body, accounting for approximately 40% of total body mass [1]. Aging is associated with a progressive decline in skeletal muscle mass, and physical performance, which contributes to sarcopenia-related functional impairment and skeletal muscle dysfunction. These changes contribute to reduced mobility, increased risk of falls and frailty, and elevated morbidity and mortality [2]. Age-related skeletal muscle deterioration is characterized by functional decline, including reduced muscle output and structural alterations such as reduced muscle mass and muscle atrophy [3]. Although functional impairment and muscle atrophy are not identical processes, they are closely interconnected and share several molecular mechanisms, including oxidative stress, mitochondrial dysfunction, chronic inflammation, and impaired protein homeostasis [4,5,6]. Mitochondrial oxidative stress plays a central role in this process, as excessive mitochondrial ROS accumulation within or around mitochondria can damage mitochondrial DNA and respiratory chain components, disrupt the redox balance, and impair oxidative phosphorylation. These mitochondrial defects compromise bioenergetic homeostasis and reduce the capacity of skeletal muscle to maintain protein turnover and cellular quality control. The resulting decline in ATP production and mitochondrial integrity control contributes to the activation of proteolytic pathways, ultimately promoting muscle atrophy and functional decline [4,5,6].

The circadian clock is a self-sustaining biological oscillator with a 24-h period that regulates a wide variety of physiological and behavioral rhythms [7,8]. This system is driven by transcriptional-translational feedback loops composed of core clock genes, including *BMAL1*, *PERs*, *CRYs*, and *RORs* [9,10,11]. It regulates diverse physiological processes including physical activity, feeding behavior, hormone secretion, sleep–wake cycles, and metabolism, thereby enabling organisms to adapt to daily environmental changes [8,11]. In skeletal muscle, circadian rhythms regulate key physiological processes, including mitochondrial energy production, mitochondrial bioenergetics, antioxidant capacity, and myogenic differentiation [12,13,14,15,16]. A properly synchronized skeletal muscle clock coordinates these processes in a time-of-day-dependent manner. During the active phase, skeletal muscle physiology is generally oriented toward movement, substrate utilization, and energy expenditure, whereas during the rest phase, it shifts toward maintenance, repair, and metabolic recovery [17]. Circadian rhythmicity in skeletal muscle declines with age, as evidenced by the reduced amplitude, phase alterations, and impaired stability of clock gene expression, leading to disrupted metabolic regulation [12,18,19]. Therefore, restoring circadian rhythmicity in aged skeletal muscle is critical for maintaining metabolic homeostasis and muscle function, and may provide a potential target for nutritional strategies aimed at mitigating sarcopenia-related decline.

Flavonoids are naturally occurring polyphenolic compounds that are widely distributed in fruits, vegetables, and medicinal plants, and are characterized by a common C6–C3–C6 carbon skeleton. They exhibit diverse biological activities, including antioxidant, anti-inflammatory, and metabolic regulatory effects [20,21]. Several flavonoids have also been reported to alleviate circadian disruption by modulating core clock gene expression and improving mitochondrial function and metabolic homeostasis [22,23,24]. Among them, hesperetin, a citrus-derived flavonoid, has been widely reported to exert antioxidant effects in oxidative stress-induced cellular and animal models, and mitochondrial protective effects in skeletal muscle cell models and aged mice [25,26,27]. However, whether hesperetin regulates skeletal muscle circadian rhythms and mitochondrial function during aging remains unclear. Previous in vitro studies have commonly used hesperetin at approximately 20 µM, a concentration shown to induce biological responses with minimal cytotoxicity [25,28]. Similarly, in vivo studies frequently administered hesperetin at approximately 100 mg/kg body weight, demonstrating its biological efficacy without any apparent adverse effects [29]. In contrast, higher concentrations or doses may induce cytotoxic or adverse responses in some experimental settings [30]. These findings support the use of hesperetin as a candidate compound for investigating the interaction between muscle aging, mitochondrial dysfunction, muscle atrophy, and time-dependent regulation.

Therefore, this study aimed to investigate the effects of hesperetin on the time-of-day-dependent clock regulation and mitochondrial function using D-galactose (D-gal)-induced senescent C2C12 myotubes in an aging-like or senescence-associated phenotype model. In addition, a dexamethasone (Dex)-induced catabolic muscle atrophy model, an established experimental model of glucocorticoid-induced skeletal muscle wasting, was used as a complementary model to evaluate whether the effects of hesperetin extend to atrophy-associated conditions.

## 2. Materials and Methods

### 2.1. D-Galactose-Induced Aging Mouse Model

**Figure 1:**
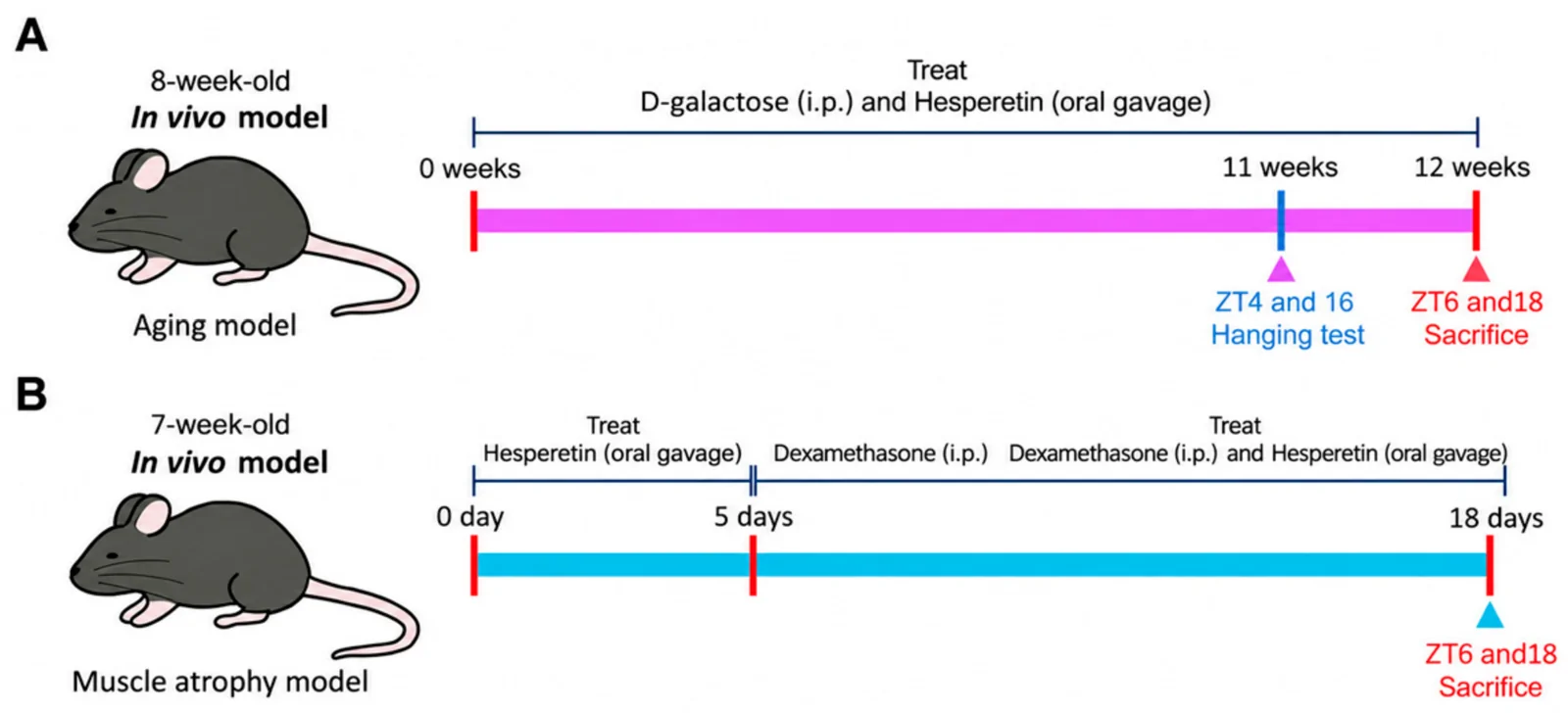
Experimental Overview. (**A**) D-gal-induced model flowchart. (**B**) Dex-induced muscle atrophy model flowchart.

D-gal-induced model is a widely used experimental model of aging-like or senescence-associated physiological alterations, particularly oxidative stress, mitochondrial impairment, and chronic inflammatory responses. Excessive D-gal exposure accelerates senescence through abnormal galactose metabolism, resulting in increased ROS generation, AGEs (advanced glycation end products) accumulation, and the activation of inflammatory signaling pathways [31,32]. Because these alterations are closely associated with skeletal muscle dysfunction under aging-like stress conditions, D-gal-induced models have been used to investigate mechanisms related to oxidative stress, mitochondrial dysfunction, and senescence-associated muscle deterioration [33]. Mice (6-week-old male C57BL/6J) were purchased from Nara Biotechnology (Seoul, Republic of Korea). A 2-week environmental acclimation period was provided to all mice before the experiments. Four animals were housed per cage and were provided free access to water and food (#D10012g, Raonbio, Yongin, Republic of Korea). The housing environment was controlled at 24 ± 1 °C and a 12 h light/dark cycle with lights turned on at 07:00. Zeitgeber time (ZT) 0 and ZT12 indicated the lights on (7:00) and lights off (19:00), respectively. The animals were randomly distributed into three groups (*n* = 8 per group). Aging was induced by intraperitoneal (i.p.) administration of D-gal (150 mg/kg body weight; Sigma-Aldrich, St. Louis, MO, USA). For 12 weeks, the animals received hesperetin (100 mg/kg body weight; Sigma-Aldrich, St. Louis, MO, USA) via oral gavage along with a D-gal injection (Figure 1A). To minimize time-of-day-dependent rhythm disturbances, D-gal and hesperetin were delivered during the light phase (ZT0–ZT2). Vehicle treatments, including intraperitoneal injections of phosphate-buffered saline (PBS) and oral gavage of corn oil, were administered to the control group animals to align with the administration routes applied to the experimental groups. Body weights and food consumption were monitored weekly. Before dissection, all animals were anesthetized using inhaled isoflurane anesthesia. A technician aware of the assignments conducted the group allocation process. Although the experimenters were aware of the group allocation, independent blind researchers performed the outcome evaluation and data analysis. All animal experiments were conducted in accordance with the guidelines for animal care and use and were approved by the Institutional Animal Care and Use Committee (IACUC) of Kongju National University (Approval date: 14 August 2024, Approval No.: KNU_2024-10).

### 2.2. Dexamethasone-Induced Muscle Atrophy Model

A Dex-induced catabolic muscle atrophy model was used as a supplementary model to further support these observed effects. Dex-induced muscle atrophy is a widely established experimental model for studying skeletal muscle wasting [34,35,36]. To further examine the observed effects in an additional muscle atrophy model, 7-week-old male C57BL/6J mice were orally administered hesperetin (100 mg/kg b.w.) dissolved in 0.5% carboxymethylcellulose (CMC) by gavage. Five days after treatment initiation, Dex (20 mg/kg/day) dissolved in DMSO and diluted with saline was administered via intraperitoneal injection for 13 days to induce skeletal muscle atrophy, while hesperetin administration was continued throughout the Dex administration period (Figure 1B). Vehicle treatments, including intraperitoneal injections of DMSO and diluted with saline and oral gavage of 0.5% CMC, were administered to the control group. All animal experiments were conducted in accordance with the guidelines for animal care and use and approved by the Institutional Animal Care and Use Committee (IACUC) of Kongju National University (Approval date: 9 March 2026, Approval No.: KNU_2026-02).

### 2.3. Muscle Fiber Staining

The soleus muscle was used for muscle fiber-type analyses. Because the soleus muscle is rich in type I and IIa oxidative fibers with elevated mitochondrial content, it was chosen for muscle fiber-type analysis to evaluate age-associated changes in mitochondrial function and oxidative metabolism.

Cryosections (12 µm thick) were produced from frozen calf tissue embedded in optimal cutting temperature (OCT) compound and stored at −80 °C before staining. After equilibration at room temperature (RT) for 10 min, sections were rinsed in PBS and TBS–Tween for 5 min per wash. The slides were immersed in a staining jar containing hot PBS and incubated in a Styrofoam box for 5 min. Following heat treatment, the slides were maintained at RT for 30 min and subsequently exposed to 1.0% Triton X-100 in 1× PBS for 2 min. Blocking was performed using 5% goat serum at RT for 1 h. Three antibodies against individual MyHC isoforms BA-D5 (type I), BF-F3 (type IIB), and SC-71 (type IIA) were mixed at a 1:85 dilution in 1% goat serum primary buffer to generate the primary antibody solution. Overnight incubation with the primary antibody solution was carried out at 4 °C. The next day, slides were rinsed sequentially in PBS and TBS–Tween for 5 min per wash. Secondary antibody incubation (1:200) was performed for 1 h. Following mounting with fluorescent mounting medium (Southern Biotech, Birmingham, AL, USA), the stained slides were imaged using a confocal microscope (ZEISS LSM 800, Carl Zeiss, Oberkochen, Germany) with ×20 or ×40 objectives.

### 2.4. Quantification of Muscle Fiber Type

Muscle fiber type composition was evaluated in the gastrocnemius, plantaris, and soleus muscle sections using muscle fiber staining. Muscle fibers were classified according to fiber type and manually counted using ImageJ software (version 1.54; NIH, Bethesda, MD, USA). All visible fibers within the analyzed fields were included, resulting in more than 3000 fibers analyzed per muscle type per mouse from non-overlapping fields. The proportion of each fiber type was calculated as a percentage of the total number of fibers analyzed.

All acquired confocal images were included in the analysis, with the number of fields of view varying among samples. All muscle fibers visible within the analyzed images were manually counted. The proportion of each fiber type was calculated as a percentage of the total number of fibers analyzed.

### 2.5. Hanging Test

One week before sacrifice, hanging tests were performed at ZT4–ZT6 and ZT16–ZT18. The mice were positioned on a grid that was gently turned over their cage. The time required to fall from the grid was recorded. Three trials were conducted per mouse and the mean value was determined. Standardized criteria were employed to reduce measurement error, with a maximum test duration of 400 s.

### 2.6. Succinate Dehydrogenase (SDH) Staining

SDH staining was performed on soleus muscles. After drying at RT for 10 min, cryosections were incubated at 37 °C for 10 min in a reaction mixture composed of 0.2 M phosphate buffer (pH 7.4), 0.1 M MgCl_2_, 0.2 M succinic acid, and 2.4 mM nitroblue tetrazolium (all from Sigma-Aldrich, St. Louis, MO, USA). After washing with deionized water and dehydration in 50% ethanol, the sections were mounted with Eukitt^®^ quick-hardening mounting medium (Sigma-Aldrich, St. Louis, MO, USA). The stained slides were imaged using a microscope (Olympus, Tokyo, Japan). The SDH-positive area was quantified as a percentage of the total muscle fiber area using ImageJ software (version 1.54). For the D-gala experiment, eight mice per group were analyzed, and fixed threshold of 130 was applied consistently to all images within this experiment. For the dex experiment, at least seven mice per group were analyzed, and fixed threshold of 170 was applied consistently to all images within this experiment.

### 2.7. Quantification of the Muscle Fiber Area

Muscle fiber area was estimated from SDH-stained images using the ImageJ software (version 1.54). Only intact muscle fibers with clearly defined boundaries were included in the analysis. For this analysis, 4–8 mice per group were included, and an average of 993 muscle fibers per group were analyzed. The horizontal and vertical diameters of the individual muscle fibers were measured, and the mean radius was calculated from these values. Muscle fiber area was then calculated using the formula πr^2^.

### 2.8. C2C12 Cell Culture, Differentiation, and Treatment

C2C12 (ATCC, #CRL-1772, Manassas, VA, USA), a mouse myoblast cell line, was grown in Dulbecco’s modified Eagle’s medium (DMEM) supplemented with 10% fetal bovine serum (FBS) and 1% penicillin/streptomycin (P/S) until they reached 90–95% confluence. Cells were then incubated in differentiation medium (DM) containing 2% horse serum for 4–6 days and 1 × insulin-transferrin-selenium (Thermo Fisher Scientific, Waltham, MA, USA) for the first 2 days. The DM was changed every other day for 7 days until the cells attained a fully differentiated state. The detailed differentiation time points are provided in the figure legends. D-gal (20 g/L; Sigma-Aldrich, St. Louis, MO, USA) was administered to skeletal muscle cells to establish a cellular aging model associated with increased oxidative stress and impaired mitochondrial redox function. Cells were treated with hesperetin (20 µM; Sigma-Aldrich, St. Louis, MO, USA) during differentiation.

### 2.9. Cell Viability Assay

Cell viability was determined using the 3-(4,5-dimethyl-2-thiazolyl)-2,5-diphenyl-2H-tetrazolium bromide (MTT) assay (Thermo Fisher Scientific, Waltham, MA, USA). C2C12 cells (1 × 10^4^ cells/well) were seeded in 96-well plates and treated with hesperetin (10 and 20 µM) for 96 h. After treatment, cells were incubated with MTT solution (M2128, Sigma-Aldrich, St. Louis, MO, USA) for 3 h at 37 °C. The supernatant was then removed, and the formazan crystals subsequently formed were dissolved in 200 µL of dimethyl sulfoxide (DMSO). The absorbance was measured at 570 nm using a microplate reader (Tecan, Männedorf, Switzerland).

### 2.10. Real-Time Polymerase Chain Reaction (PCR) Analysis

In vitro, differentiated C2C12 cells were collected every 4 h for 24 h to evaluate circadian oscillatory patterns. Calf muscles harvested at ZT6 and ZT18 were pulverized in vivo using a tissue pulverizer. Total RNA was isolated using RiboEx RNAsol reagent (GeneAll, Seoul, Republic of Korea), followed by reverse transcription of 2 µg of RNA into cDNA using a cDNA synthesis kit (Thermo Fisher Scientific). Gene expression levels were determined by real-time PCR using RealAmp 2× qPCR Master Mix high ROX (GeneAll) on a QuantStudio 5 Real-Time PCR System (Thermo Fisher Scientific, Waltham, MA, USA). Each sample was analyzed in duplicate. Relative mRNA levels were determined using the 2^−ΔΔCt^ method, with GAPDH as an internal control. The primer sequences are listed in Table 1.

### 2.11. Hematoxylin and Eosin (H&E) Staining

C2C12 cells cultured in DM for 2, 4, and 6 d were morphologically confirmed by myotube formation using an H&E staining kit (Abcam, Cambridge, UK). The differentiated C2C12 cells were fixed in 4% paraformaldehyde (Sigma-Aldrich, St. Louis, MO, USA) and stained with H&E for cell morphological evaluation. Histological analyses were performed in triplicate for each group. Images were captured using a Leica DMi1 microscope (Leica Microsystems, Wetzlar, Germany). The differentiation ratio was quantified using ImageJ software (version 1.54).

### 2.12. Immunofluorescence (IF) Staining

Differentiated C2C12 myotubes grown on coverslips, washed with PBS and fixed in 4% paraformaldehyde in PBS at RT for 10 min. After three washes with PBS, cells were treated with 0.1% Triton X-100 (GeneAll) for 10 min and subsequently blocked in 5% goat serum at RT for 60 min. Overnight incubation at 4 °C was performed with a skeletal muscle myosin antibody (sc-32732, Santa Cruz Biotechnology, Dallas, TX, USA). After three washes with PBS, cells were incubated with goat anti-mouse IgG Alexa Fluor 488 (Thermo Fisher Scientific, Waltham, MA, USA) at RT for 60 min. Images were acquired using a confocal microscope (Zeiss). To evaluate nonspecific interactions and background fluorescence, no primary antibody or isotype control samples were used as negative controls.

### 2.13. Senescence-Associated β-Galactosidase (SA-β-Gal) Staining

Five days after differentiation, SA-β-gal staining was performed on differentiated C2C12 myotubes with D-gal-induced senescence using a Senescence β-Galactosidase Staining Kit (Cell Signaling Technology, Danvers, MA, USA) according to the manufacturer’s instructions. Images were acquired using the Leica DMi1 microscope (Leica Microsystems). SA-β-gal–positive cells were quantified by counting both total and positively stained cells using ImageJ software (version 1.54), and the ratio of SA-β-gal-positive cells was calculated from the total cell number.

### 2.14. Intracellular Reactive Oxygen Species (ROS) Detection

To assess intracellular oxidative stress-associated fluorescence, C2C12 cells were treated with D-gal and hesperetin throughout the 5-day differentiation period and then incubated with 25 µM 2′,7′-dichlorodihydrofluorescein diacetate (DCF-DA) for 30 min at 37 °C. The fluorescence intensity was recorded using a microplate reader (Tecan).

### 2.15. Enzymatic Activities

Commercial assay kits (Cayman Chemical, Ann Arbor, MI, USA) were used to determine intracellular Malondialdehyde (MDA) levels and the activities of superoxide dismutase (SOD), Catalase (CAT), and glutathione peroxidase (GPx), in accordance with the manufacturer’s protocols. They were normalized to total protein concentration determined by BCA protein assay. The results were expressed relative to total protein content and normalized to the control group where appropriate.

### 2.16. pMitoTimer Transfection and Imaging

The *pMitoTimer* reporter plasmid (#52659; Addgene, Watertown, MA, USA) was introduced into C2C12 myoblasts by transfection using Lipofectamine 3000 (Thermo Fisher Scientific, Waltham, MA, USA) according to the manufacturer’s guidelines. Cells were seeded in 6-well plates and transfected at approximately 50% confluence. For each well, 2 µg of plasmid DNA was mixed with P3000 reagent and Lipofectamine 3000 in DMEM. After overnight transfection, the medium was replaced and cells were treated with D-gal with or without hesperetin for 30 h. Mitochondrial fluorescence was imaged using a confocal microscope (Zeiss) with ×20 objective. Three wells per experimental group were analyzed, and a total of 20 fields of view were imaged across the three wells. Red and green fluorescence intensities were quantified using ImageJ software (version 1.54), and the mitochondrial oxidative status was expressed as the green/red fluorescence intensity ratio.

### 2.17. MitoTracker Staining

Four days after differentiation, MitoTracker Red CMXRos (Thermo Fisher Scientific, Waltham, MA, USA) was added to the DM of differentiated C2C12 cells at a final concentration of 50 nM for 30 min to label the mitochondria. After staining, the cells were rinsed twice with PBS and fixed with 4% paraformaldehyde (PFA). The cells were mounted using a mounting solution containing 4′,6-diamidino-2-phenylindole (DAPI). Fluorescence images were captured using a confocal microscope (Zeiss), and fluorescence intensity was quantified using a microplate reader (Tecan).

### 2.18. ATP Measurement

To measure ATP levels, differentiated C2C12 cells were gently rinsed three times with PBS. Intracellular ATP levels were quantified using an ATP assay kit (Abcam, Cambridge, UK) following the manufacturer’s instructions. ATP levels were normalized to total protein concentration. The results were expressed relative to total protein content and normalized to the control group where appropriate.

### 2.19. Mitochondrial Membrane Potential (MMP) Measurement

The MMP was determined using the cationic fluorescent dye JC-1 (Thermo Fisher Scientific, Waltham, MA, USA). In healthy mitochondria with preserved membrane potential, JC-1 assembles into red-emitting aggregates (Ex/Em = 560/595 nm), whereas the loss of membrane potential maintains the dye in a green fluorescent monomeric state (Ex/Em = 485/535 nm). Cells were incubated with 10 µM JC-1 for 20 min at 37 °C in the dark, followed by fluorescence detection using a microplate reader (Tecan). Mitochondrial membrane potential (MMP) was quantified by calculating the fluorescence intensity ratio of red (aggregates) to green (monomers).

### 2.20. Quantification and Statistical Analysis

Results are expressed as mean ± standard error of the mean (SEM). Statistical testing was performed using Student’s *t*-test and one-way or two-way analysis of variance (ANOVA), followed by Tukey’s post hoc multiple comparison test. Effect sizes for selected in vivo outcomes were calculated as partial eta squared (ηp^2^), using the formula ηp^2^ = SS_effect/(SS_effect + SS_residual). Post hoc power was estimated for representative in vivo outcomes, including hanging test and SDH staining, based on the observed effect size, total sample size, and α = 0.05. All analyses were performed using the Prism 10 software (GraphPad ver. 11.1.0, San Diego, CA, USA). Statistical significance was set at *p* < 0.05. 

## 3. Results

### 3.1. Hesperetin Improves Muscle Performance in a D-Gal–Induced Aging Mouse Model

**Figure 2:**
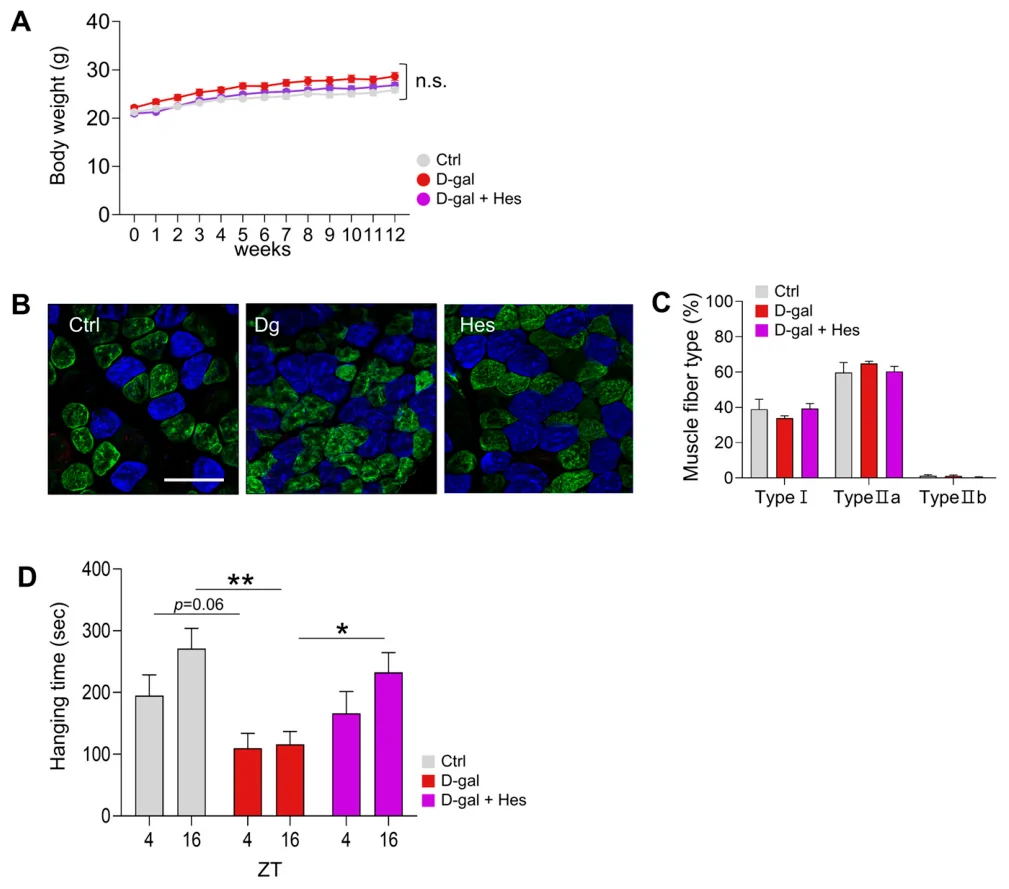
The Effect of Hesperetin on Skeletal Muscle Function in a D–Gal–Induced Mouse Model. (**A**) Mouse body weight analysis (*n* = 8/group). All values are presented as mean ± SEM. One-way ANOVA with Tukey’s multiple comparisons. (**B**) Confocal microscopy analysis of muscle fiber type in soleus muscle (Blue: Type I, green: Type IIA). 20× magnification. Scale bar: 100 μm. (**C**) Quantification of the proportion of muscle fiber types in soleus muscle. All values are presented as mean ± SEM. One-way ANOVA with Tukey’s multiple comparisons. (**D**) Hanging time (s) at ZT4 and ZT16 (*n* = 8/group). All values are presented as mean ± SEM. Two-way ANOVA with Tukey’s multiple comparisons. * *p* < 0.05, ** *p* < 0.01. n.s.: not significant; Ctrl; Control; D-gal (Dg); D-galactose, Hes; Hesperetin, ZT; Zeitgeber Time.

No significant differences in body weight were observed among the groups during the weekly monitoring (Figure 2A). Histological alterations in muscle fiber composition were evaluated by muscle fiber-type staining in the soleus, plantaris, and gastrocnemius muscles (Figure 2B,C). No significant differences in the muscle fiber composition were observed among the groups for any of the examined muscles.

To evaluate the effects of hesperetin on skeletal muscle functional output in D-gal-induced aging mice, hanging test was performed in the control, D-gal, and D-gal + Hes groups (Figure 2D). In the control group, muscle function exhibited diurnal rhythmicity, with a significantly higher performance at ZT16, corresponding to the active phase, than at ZT4. D-gal treatment reduced the overall motor performance and attenuated the diurnal variation in muscle function compared to the control group (*p* < 0.01). Specifically, at ZT16, hanging time decreased by 57.2% in the D-gal group compared with the control group (Control: 271.2 ± 32.69 s vs. D-gal: 116 ± 20.83 s, mean ± SEM, *p* < 0.01). Hesperetin administration (100 mg/kg body weight) increased hanging time by 100.5% compared with the D-gal group (D-gal: 116 ± 20.83 s vs. Hesperetin: 232.6 ± 32.03 s, mean ± SEM, *p* < 0.05). For the hanging test, two-way ANOVA showed significant group effect (F(2,42) = 8.382, *p* = 0.0009, partial η^2^ = 0.285). Post hoc power analysis based on this group effect indicated adequate statistical power (achieved power = 0.973 at α = 0.05). These results suggest that hesperetin improves D-gal-induced impairment of muscle performance and partially restores the diurnal pattern of skeletal muscle function.

### 3.2. Hesperetin Restores Core Clock Gene Oscillations in a D-Gal–Induced Aging Mouse Model

**Figure 3:**
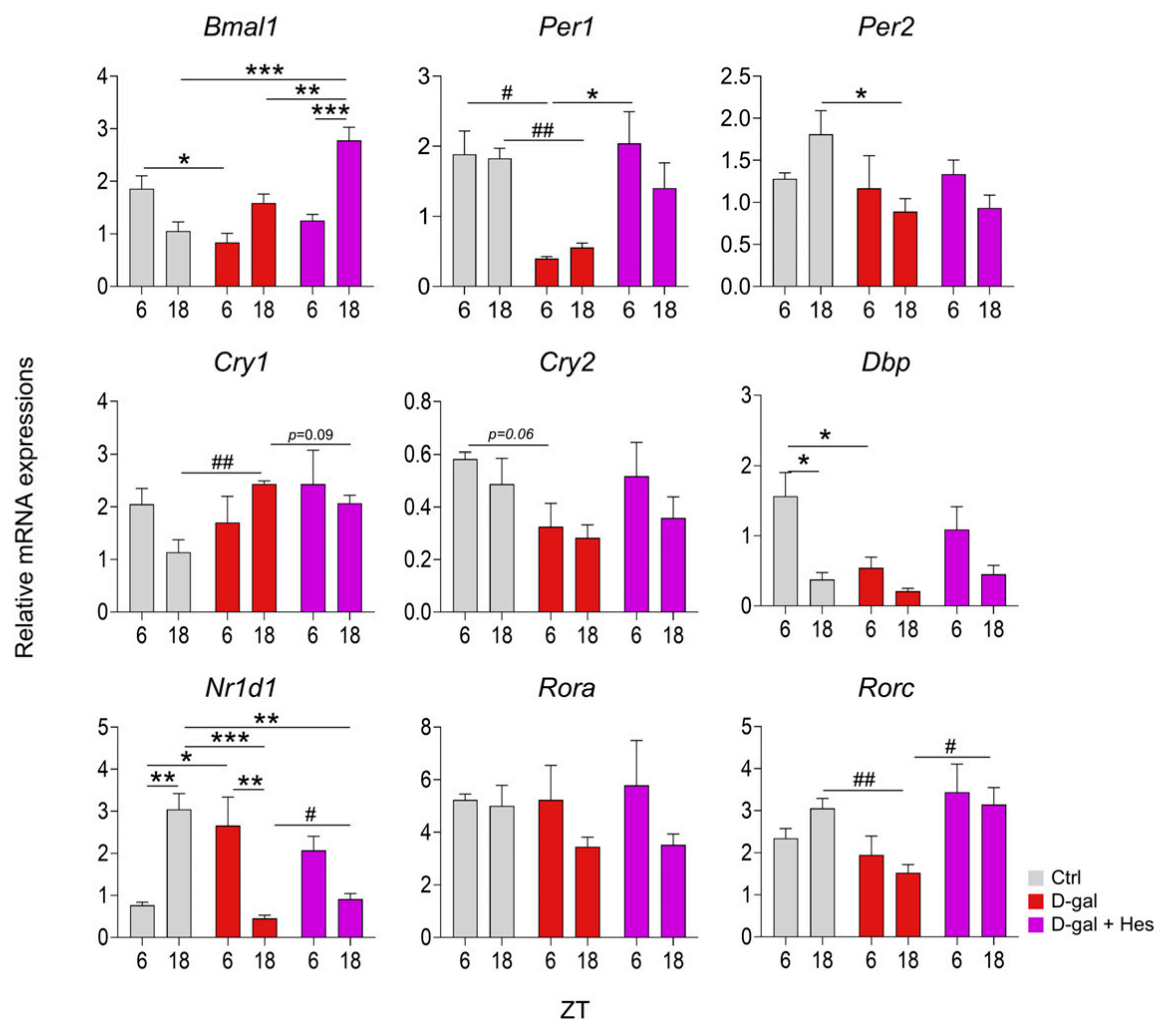
Hesperetin Restores Diurnal Change in Core Clock Genes in a D–Gal–Induced Mouse Skeletal Muscle. Core clock gene expression in calf tissues at ZT6 and ZT18 was analyzed by real-time qPCR. All the values are presented as mean ± SEM (*n* = 3–4/group/time point). Two-way ANOVA with Tukey’s multiple comparisons and Welch’s *t*-test. * *p* < 0.05, ** *p* < 0.01, *** *p* < 0.001 (Two-way ANOVA). # *p* < 0.05, ## *p* < 0.01 (Welch’s *t*-test). Ctrl; Control, D-gal; D-galactose, Hes; Hesperetin, ZT; Zeitgeber Time.

To determine whether Hesperetin modulates circadian clock gene expression in D-gal-induced muscle aging, expression levels of core clock genes were analyzed in skeletal muscle collected at ZT6 and ZT18 (Figure 3). Real-time PCR was performed to assess the expression of core clock genes in calf muscles. D-gal treatment markedly disrupted the diurnal expression patterns of several core clock genes compared to the control group. In the control group, basic helix-loop-helix ARNT like 1 (*Bmal1*), period circadian clock 2 (*Per2*), cryptochrome circadian regulator 1 (*Cry1*), nuclear receptor subfamily 1 group D member 1 (*Nr1d1*), and RAR-related orphan receptor gamma (*Rorc*) showed ZT6–ZT18 differences in expression. D-gal treatment altered these time-of-day-dependent expression patterns, with *Bmal1*, *Per2*, *Cry1*, *Nr1d1*, and *Rorc* showing expression patterns opposite to those observed in the control group. Although RAR-related orphan receptor alpha (*Rora*) expression did not show a significant ZT6–ZT18 difference in the control group, D-gal treatment induced de novo diurnal changes.

Hesperetin treatment partially modulated the altered expression of the selected clock genes. At ZT18, hesperetin treatment significantly increased *Bmal1* expression by 74.9% compared with the D-gal group (mean ± SEM, *p* < 0.01). Period circadian clock 1 (*Per1)* mRNA expression was reduced by 78.9% and 69.6% at ZT6 and ZT18, respectively, in D-gal group compared with control group. Hesperetin treatment elevated *Per1* expression by 414.2% at ZT6 and 152.9% at ZT18 relative to the D-gal group (mean ± SEM, *p* < 0.05). In contrast, *Per2*, cryptochrome circadian regulator 2 (*Cry2*), D site albumin promoter binding protein (*Dbp*), and *Rora* expression levels did not differ significantly between the D-gal- and hesperetin-treated groups. Overall, these results suggested that hesperetin partially restored and normalized the disrupted circadian expression patterns of core clock genes during D-gal-induced muscle aging.

### 3.3. Hesperetin Improves Mitochondrial-Associated Indicators in a D-Gal–Induced Aging Mouse Model

**Figure 4:**
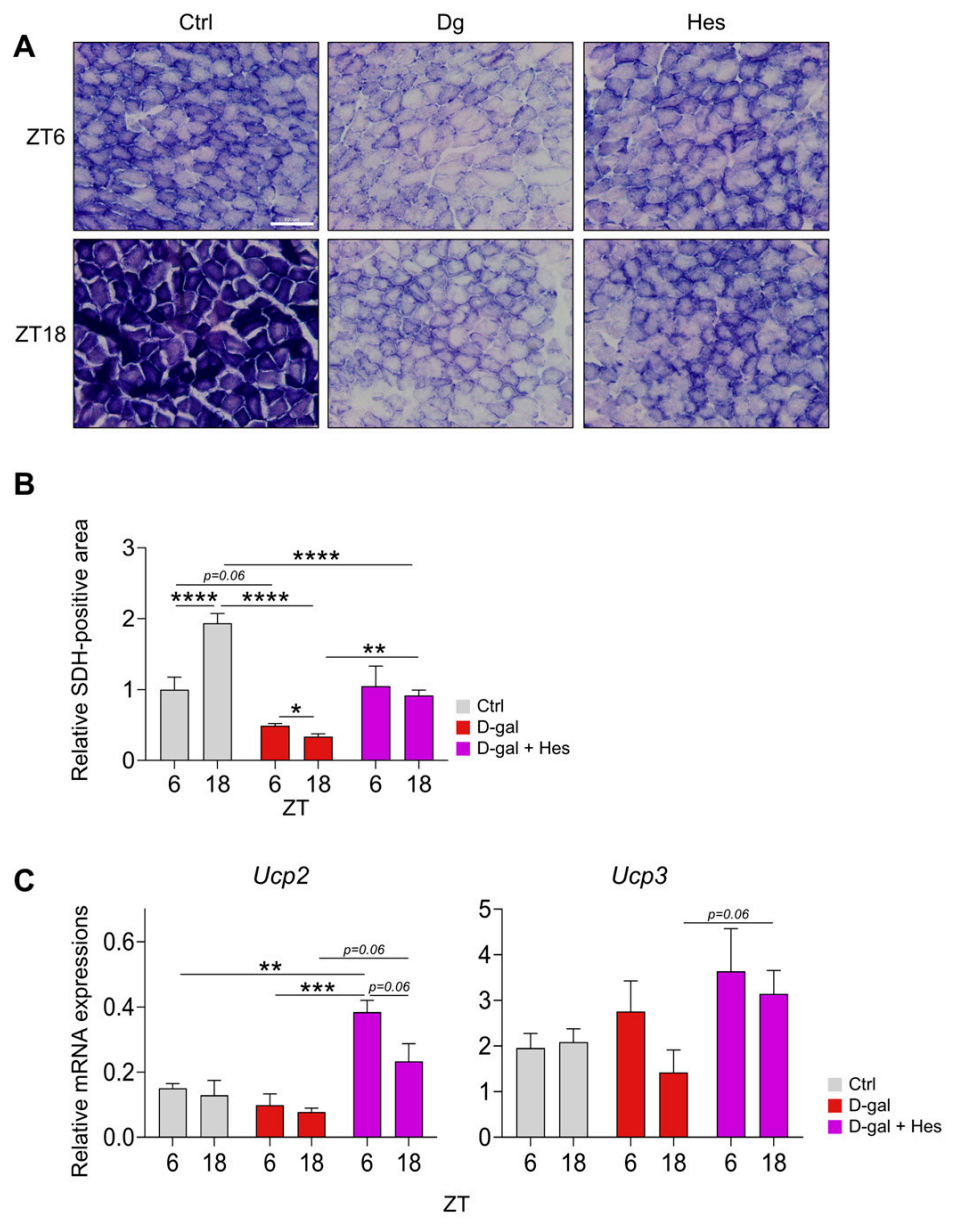
Hesperetin Enhances Mitochondrial Oxidative Capacity and Mitochondrial Function-Related Gene Expressions in a D–Gal–Induced Mouse Model. (**A**) Succinate dehydrogenase (SDH) staining in soleus muscle. 20× magnification. Scale bar: 100 μm. (**B**) Quantification of the proportion of SDH-positive fibers in the soleus muscle at ZT6 and ZT18 (Threshold: 130). SDH-positive area was quantified as a percentage of the total muscle fiber area, and values were normalized to the mean value of the control group at ZT6, which was set to 1. Data are shown as mean ± SEM. Two-way ANOVA with Tukey’s multiple comparisons. (**C**) Mitochondrial function–related gene expressions in calf tissues at ZT6 and ZT18 were analyzed by real-time qPCR. All values are presented as mean ± SEM (*n* = 3–4/group/time point). Two-way ANOVA with Tukey’s multiple comparisons. * *p* < 0.05, ** *p* < 0.01, *** *p* < 0.001, **** *p* < 0.0001. Ctrl; Control, D-gal (Dg); D-galactose, Hes; Hesperetin, ZT; Zeitgeber Time.

SDH staining was performed to assess SDH-positive area as an indicator of mitochondrial oxidative enzyme activity in skeletal muscle (Figure 4A). In the control group, SDH-positive area was significantly 93.6% higher at ZT18 than at ZT6, indicating a clear diurnal variation (Figure 4B). In contrast, D-gal treatment markedly reduced the SDH-positive area by 51.0% at ZT6 and 82.5% at ZT18 compared with the corresponding control group, and altered the normal diurnal pattern, showing an anti-phase response compared to the control group (*p* < 0.0001). Two-way ANOVA showed a significant group effect (F(2,18) = 24.13, *p* < 0.0001, partial η^2^ = 0.728, post hoc power > 0.999) and a significant group × time interaction (F(2,18) = 8.368, *p* = 0.0027, partial η^2^ = 0.482, post hoc power = 0.979). Hesperetin treatment significantly mitigated the D-gal-induced reduction in the SDH-positive area and partially restored the diurnal pattern of mitochondrial oxidative capacity (*p* < 0.01).

To further assess the expression of mitochondrial function-related genes, including uncoupling protein 2 (*Ucp2*), and uncoupling protein 3 (*Ucp3*), was analyzed in calf muscle at ZT6 and ZT18 using real-time qPCR (Figure 4C). Hesperetin treatment increased the diurnal amplitude of *Ucp2* expression, suggesting a partial recovery of rhythmicity after D-gal-induced attenuation. The expression levels of *Ucp2*, which were reduced by D-gal treatment, were significantly increased by 288.9% at ZT6 and 200.0% at ZT18 following hesperetin administration compared with the D-gal group (mean ± SEM, *p* < 0.001 and *p* = 0.06). *Ucp3* showed a similar increasing trend in diurnal amplitude after hesperetin treatment, although this change was not statistically significant (*p* = 0.06). Collectively, these findings suggest that hesperetin improves SDH-positive area and modulates mitochondrial function-related gene expression in D-gal-treated mice.

### 3.4. Hesperetin Protects Against Dex-Induced Skeletal Muscle Atrophy

**Figure 5:**
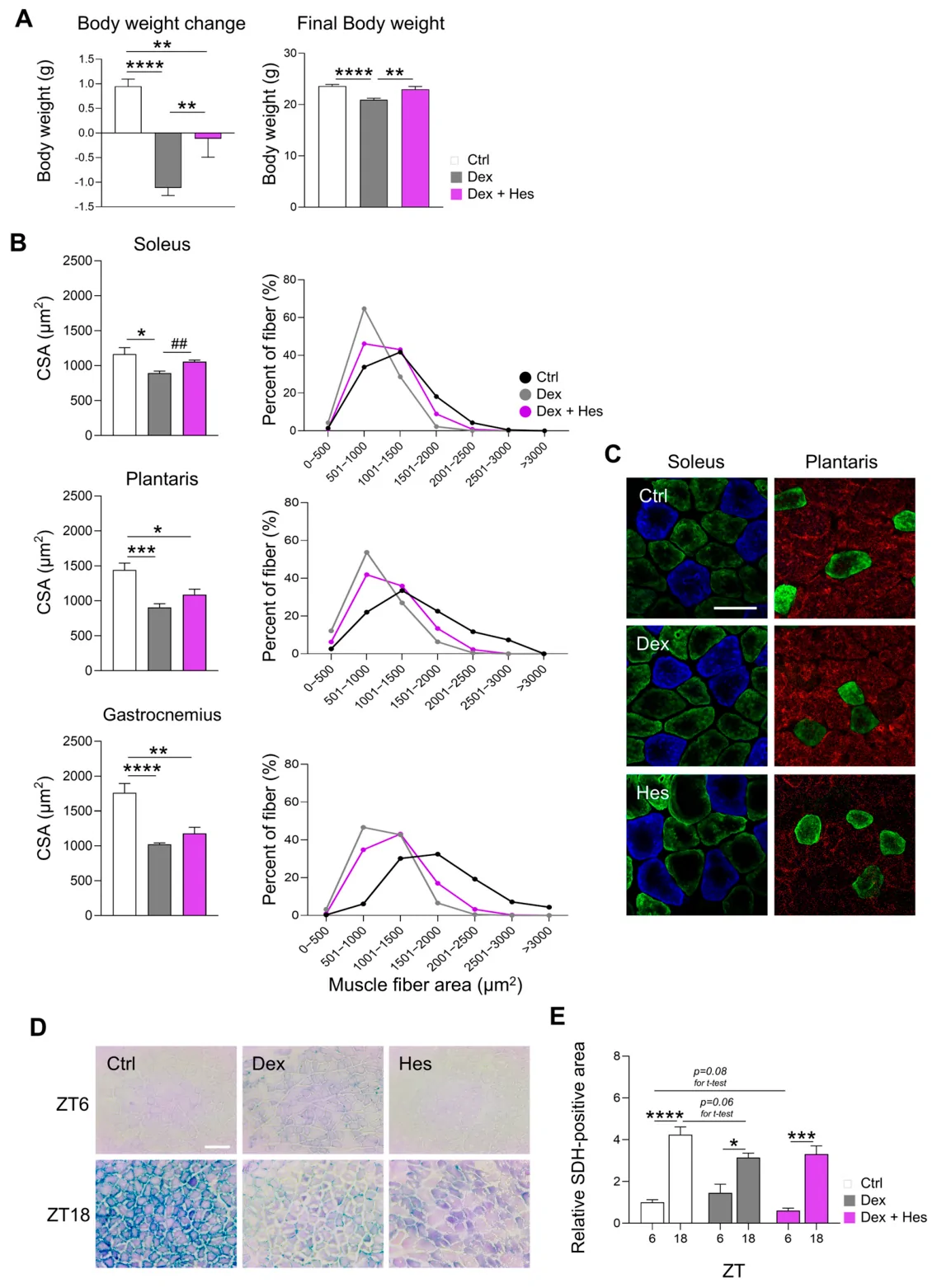
Hesperetin Attenuates Skeletal Muscle Atrophy in a Dex–Induced Atrophy Mouse Model. (**A**) Mouse body weight analysis (*n* = 8/group). All values are presented as mean ± SEM. One-way ANOVA with Tukey’s multiple comparisons. (**B**) Quantification of the muscle fiber area in calf tissues by SDH staining image. CSA = cross-sectional area. (**C**) Confocal microscopy analysis of muscle fiber type in soleus and plantaris muscle (Blue: Type I, green: Type IIA, red: Type IIB). 40× magnification. Scale bar: 50 μm. (**D**) Succinate Dehydrogenase (SDH) staining in soleus muscle. 20× magnification. Scale bar: 100 μm. (**E**) Quantification of the proportion of SDH-positive fibers in soleus muscle at ZT6 and ZT18 (Threshold: 170). Values were normalized to the mean value of the control group at ZT6, which was set to 1. Data are shown as mean ± SEM. Two-way ANOVA with Tukey’s multiple comparisons. * *p* < 0.05, ** *p* < 0.01, *** *p* < 0.001, **** *p* < 0.0001 (Two-way ANOVA). ## *p* < 0.01 (Welch’s *t*-test). Dex: Dexamethasone, Hes: Hesperetin, ZT: Zeitgeber Time.

To evaluate the effects of hesperetin on Dex-induced muscle atrophy, changes in body weight, muscle fiber characteristics, mitochondrial oxidative capacity, and the expression of muscle atrophy and differentiation-related genes were assessed in the control, Dex, and Dex + Hes groups. Body weight increased by 0.95 ± 0.15 g in the control group but decreased by 1.11 ± 0.16 g in the Dex group (*p* < 0.0001). Hesperetin treatment attenuated Dex-induced body weight loss, with a body weight change of −0.11 ± 0.38 g (mean ± SEM, *p* < 0.01 vs. Dex). Final body weight was significantly reduced by 11.3% in the Dex group compared with the control group (*p* < 0.0001), whereas hesperetin treatment significantly restored body weight by 9.8% compared with the Dex group (*p* < 0.01) to a level comparable to that in the control group (Figure 5A). The muscle fiber area was quantified using SDH-stained images to evaluate the degree of muscle atrophy (Figure 5B). Compared with the control group, mean muscle fiber CSA was reduced by 42.0%, 37.0%, and 23.1% in the gastrocnemius, plantaris, and soleus muscles, respectively, in the Dex group. Hesperetin treatment increased mean CSA by 15.0%, 20.1%, and 17.9% in the gastrocnemius, plantaris, and soleus muscles, respectively, compared with the Dex group. Consistent with these changes, the Dex group showed shift toward smaller fiber sizes, particularly with a higher proportion of fibers in the 501–1000 μm^2^ range, in the gastrocnemius, plantaris, and soleus muscles. This difference was particularly evident in the gastrocnemii. Hesperetin showed a tendency to restore this effect, with a significant improvement observed in the soleus muscle. Muscle fiber type distribution in calf muscle was assessed using muscle fiber staining (Figure 5C). No significant differences in the fiber type were observed among the soleus and plantaris. Dex treatment resulted in a decreased proportion of SDH-positive fibers by 25.8% relative to the control group at ZT18 (Figure 5D,E). Two-way ANOVA showed a significant time effect (F(1,17) = 97.34, *p* < 0.0001, partial η^2^ = 0.851, post hoc power > 0.999). The group effect was not significant, and the time × group interaction showed only a trend toward significance (F(2,17) = 3.274, *p* = 0.0627).

**Figure 6:**
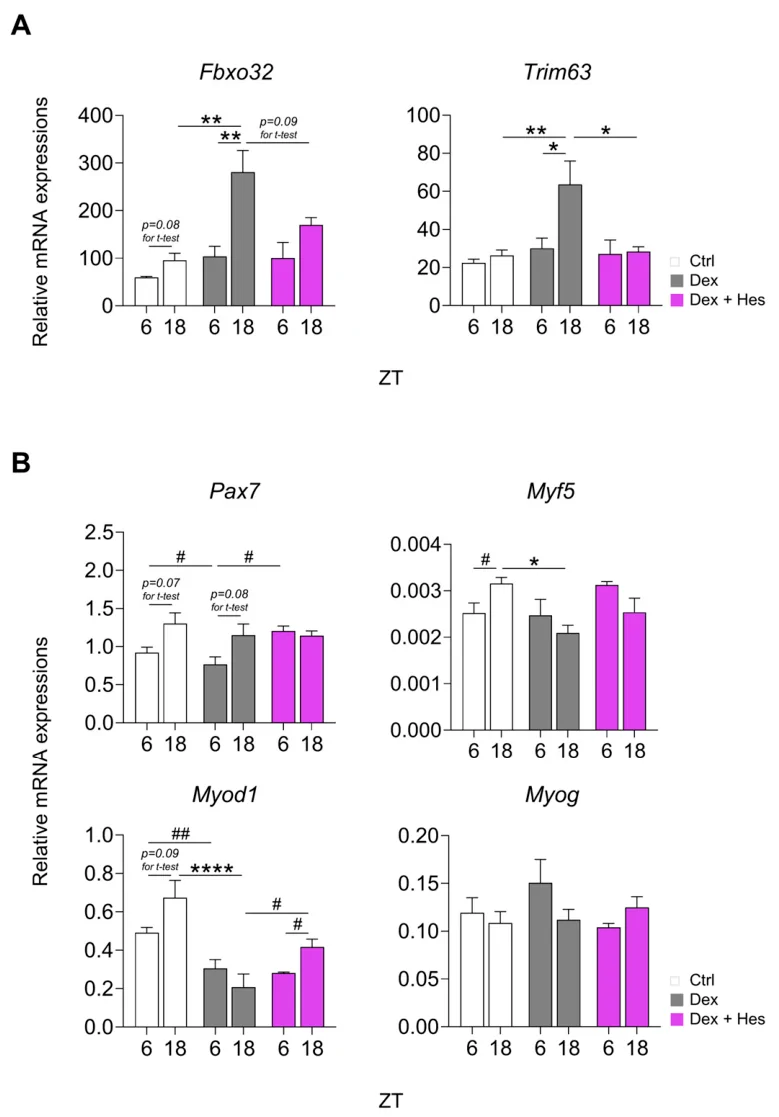
Diurnal expression of muscle atrophy and differentiation genes in a Dex–Induced Atrophy Mouse Model. (**A**,**B**) Expression of genes related to muscle atrophy (**A**) and muscle differentiation (**B**) in calf tissues at ZT6 and ZT18 was analyzed by real-time qPCR. All values are presented as mean ± SEM (*n* = 3–4/group/time point). Two-way ANOVA with Tukey’s multiple comparisons and Welch’s *t*-test. * *p* < 0.05, ** *p* < 0.01, **** *p* < 0.0001 (Two-way ANOVA). # *p* < 0.05, ## *p* < 0.01 (Welch’s *t*-test). Ctrl; Control, Dex; Dexamethasone, Hes; Hesperetin, ZT; Zeitgeber Time.

The expression levels of muscle atrophy-related genes, F-box protein 32 (*Fbxo32*) and tripartite motif-containing 63 (*Trim63*), and differentiation-related genes, paired box 7 (*Pax7*), myogenic factor 5 (*Myf5*), myogenic differentiation 1 (*Myod1*), and myogenin (*Myog*), in calf tissues were analyzed across time-of-day-dependent points (Figure 6A,B). The ZT6–ZT18 differences in *Fbxo32* and *Trim63* expression were in the control group, respectively, increased to in the Dex group, and decreased to following hesperetin treatment (Figure 6A). Dex treatment reduced *Pax7* mRNA expression by 17.0% at ZT6 compared with the control group (*p* < 0.05), whereas hesperetin treatment increased its expression by 57.3% compared with the Dex group (*p* < 0.05; Figure 6B). At ZT18, Dex treatment reduced *Myf5* mRNA expression by 34.0% compared with the control group (*p* < 0.05). Similarly, *Myod1* expression was reduced by 69.0% following Dex treatment compared with the control group (*p* < 0.0001), whereas hesperetin treatment increased its expression by 95.0% compared with the Dex group (*p* = 0.05). Notably, the ZT6–ZT18 difference in *Myod1* expression observed in the control group was lost following Dex treatment but reappeared following hesperetin treatment. No significant differences in *Myog* expression were observed between the groups. Overall, these findings suggest that hesperetin restores muscle atrophy in a mouse model of Dex-induced atrophy.

### 3.5. Hesperetin Regulates Circadian Core Clock Gene Oscillations Disrupted by D-Gal-Induced Senescence in Differentiated C2C12 Cells

**Figure 7:**
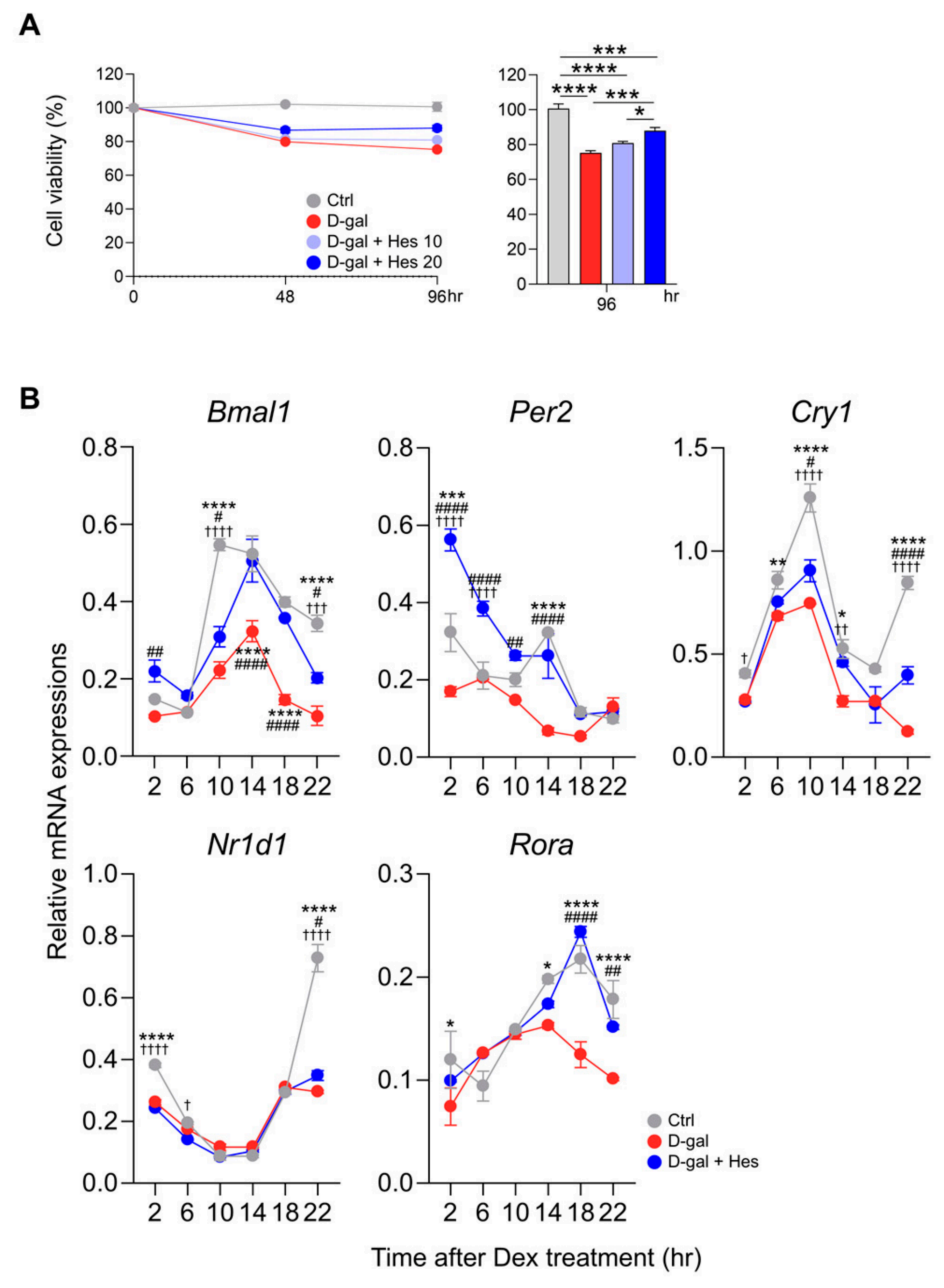
Circadian Expression of Core Clock Genes Following Hesperetin Treatment in Differentiated C2C12 Cells. (**A**) MTT assay was performed using hesperetin at concentrations of 10 and 20 µM, depending on the experimental time points. One-way ANOVA with Tukey’s multiple comparisons. (**B**) Core clock gene expression in C2C12 cells at day 4 after differentiation was analyzed by real-time qPCR. All the values are presented as mean ± SEM every 4 h over a 24-h period following Dex synchronization (*n* = 3/group/time point). *: Crtl vs. D-gal, #: D-gal vs. Hes, †: Ctrl vs. Hes. Two-way ANOVA with Tukey’s multiple comparisons. * *p* < 0.05, ** *p* < 0.01, *** *p* < 0.001, **** *p* < 0.0001, # *p* < 0.05, ## *p* < 0.01, #### *p* < 0.0001, † *p* < 0.05, †† *p* < 0.01, ††† *p* < 0.001, †††† *p* < 0.0001. D-gal; D-galactose, Hes; Hesperetin.

To determine the appropriate hesperetin concentration for subsequent experiments, cell viability following D-gal and hesperetin treatment was assessed using the MTT assay (Figure 7A). Compared with the control group, D-gal treatment significantly reduced cell viability by 21.7% at 48 and 25.1% at 96 h (*p* < 0.0001). Conversely, hesperetin treatment tended to improve cell viability in a dose-dependent manner at 10 and 20 µM. At 96 h, 20 µM hesperetin treatment markedly improved cell viability by 16.9% relative to the D-gal group (*p* < 0.001). Therefore, hesperetin at 20 µM was used for subsequent experiments.

To examine whether hesperetin modulates D-gal-induced alterations in circadian clock gene expression, the oscillatory expression patterns of core clock genes (*Bmal1*, *Per2*, *Cry1*, *Nr1d1*, and *Rora*) were assessed in differentiated C2C12 cells at 4-h intervals over 24 h (Figure 7B). D-gal-induced senescence markedly reduced the oscillation of core clock genes relative to the control group. In particular, an anti-phase oscillatory pattern was observed for *Per2* in the D-gal group compared to that in the control group. Disrupted oscillations were restored by hesperetin treatment. Specifically, hesperetin treatment normalized the time-of-day-dependent expression pattern of *Per2* and restored its diurnal expression to control levels. Hesperetin treatment markedly increased the expressions of *Bmal1*, *Per2*, and *Rora*. Taken together, these results indicate that hesperetin mitigates D-gal-induced disruptions in circadian clock gene rhythms by enhancing rhythmic amplitude and recovering phase alterations.

### 3.6. Hesperetin Modulates Muscle Cell Differentiation and Circadian Rhythmicity of Myogenic Genes in D-Gal–Induced Senescence

**Figure 8:**
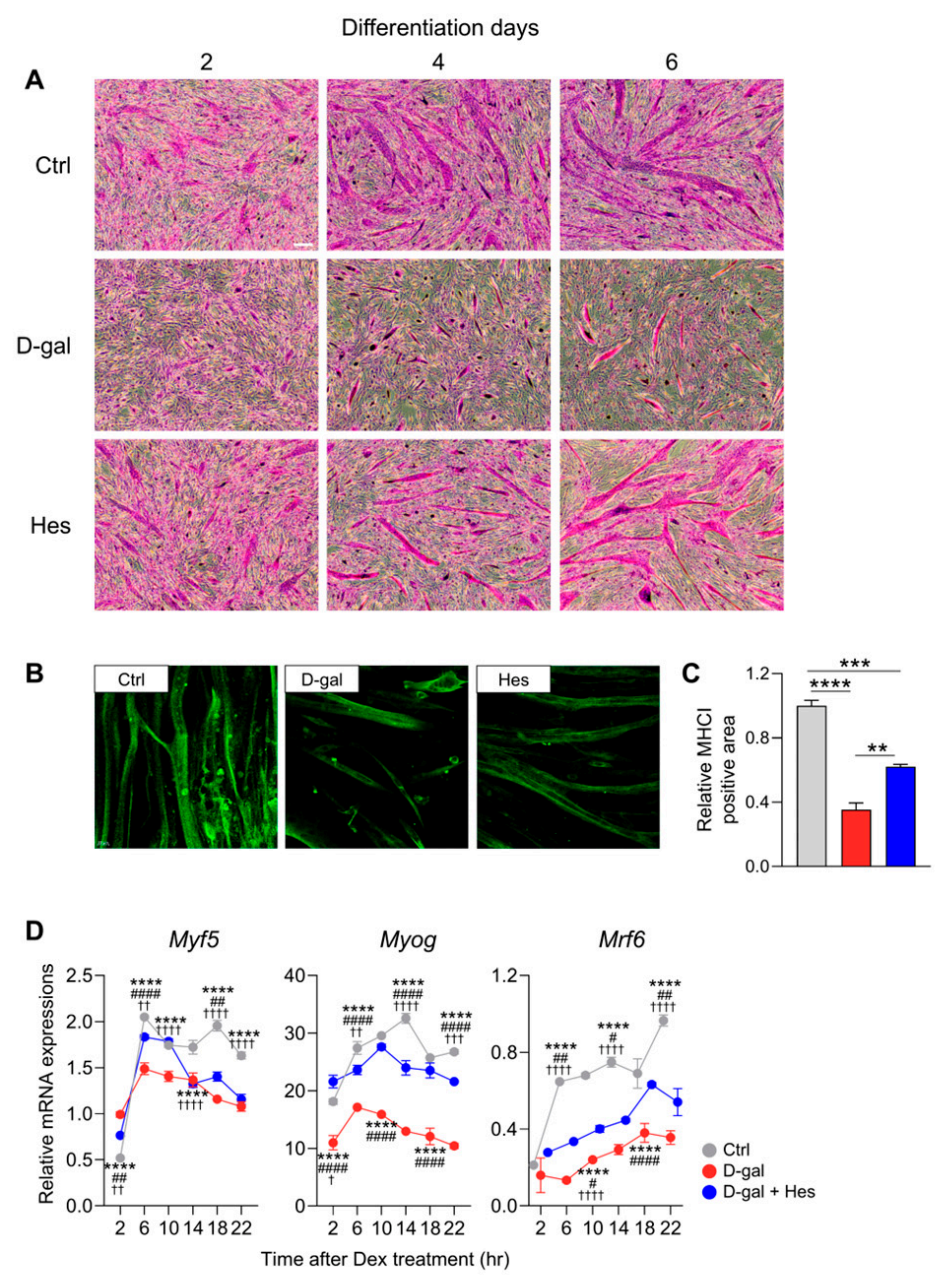
Hesperetin Improves Myotube Formation and Restores Circadian Phase Modulation of Differentiation-Related Genes in a D-Gal–Induced Senescence. (**A**) H&E staining of differentiated cells at days 2, 4, and 6. 10× magnification. Scale bar = 100 µm. (**B**) Immunofluorescence staining of MHCI. (**C**) Quantification of MCHI Immunofluorescence staining. Fluorescence intensity was quantified and expressed relative to the control group. All values are presented as mean ± SEM. One-way ANOVA with Tukey’s multiple comparisons. (**D**) Muscle differentiation-related gene expression in C2C12 cells at day 4 after differentiation was analyzed by real-time qPCR. All the values are presented as mean ± SEM every 4 h over a 24-h period following Dex synchronization (*n* = 3/group/time point). *: Crtl vs. D-gal, #: D-gal vs. Hes, †: Ctrl vs. Hes. Two-way ANOVA with Tukey’s multiple comparisons. ** *p* < 0.01, *** *p* < 0.001, **** *p* < 0.0001, # *p* < 0.05, ## *p* < 0.01, #### *p* < 0.0001, † *p* < 0.05, †† *p* < 0.01, ††† *p* < 0.001, †††† *p* < 0.0001. Ctrl; Control, D-gal; D-galactose, Hes; Hesperetin.

To assess the effects of D-gal-induced senescence on muscle cell histology, H&E staining was performed 2, 4, and 6 d after differentiation (Figure 8A). To visualize skeletal muscle myosin heavy chain (MHC), immunofluorescence staining was performed (Figure 8B). Compared with the control, D-gal treatment impaired myotube formation, reducing the MHC-positive area by approximately 64%. However, hesperetin treatment enhanced myotube differentiation, increasing the MHC-positive area by approximately 69% compared with the D-gal group (Figure 8C). D-gal treatment reduced myotube formation compared to the control, whereas hesperetin treatment restored myosin expression and improved the differentiation capacity. The expression levels of major myogenic regulatory factors, including *Myf5*, *Myog*, and myogenic factor 6 (*Myf6*), were examined across circadian time points to analyze the molecular mechanisms underlying myogenic differentiation (Figure 8D). Cellular senescence induced by D-gal significantly decreased both the overall expression and diurnal amplitude of these genes relative to the control group. Hesperetin treatment partially restored *Myf5* expression to control levels and significantly increased the circadian oscillations of *Myog* and *Myf6* expression. These findings suggest that hesperetin improves and stabilizes the circadian rhythm of muscle differentiation-associated genes and enhances the differentiation capacity in D-gal-induced senescence.

### 3.7. Hesperetin Attenuates Oxidative Stress-Related Markers in D-Gal-Induced Cellular Senescence

**Figure 9:**
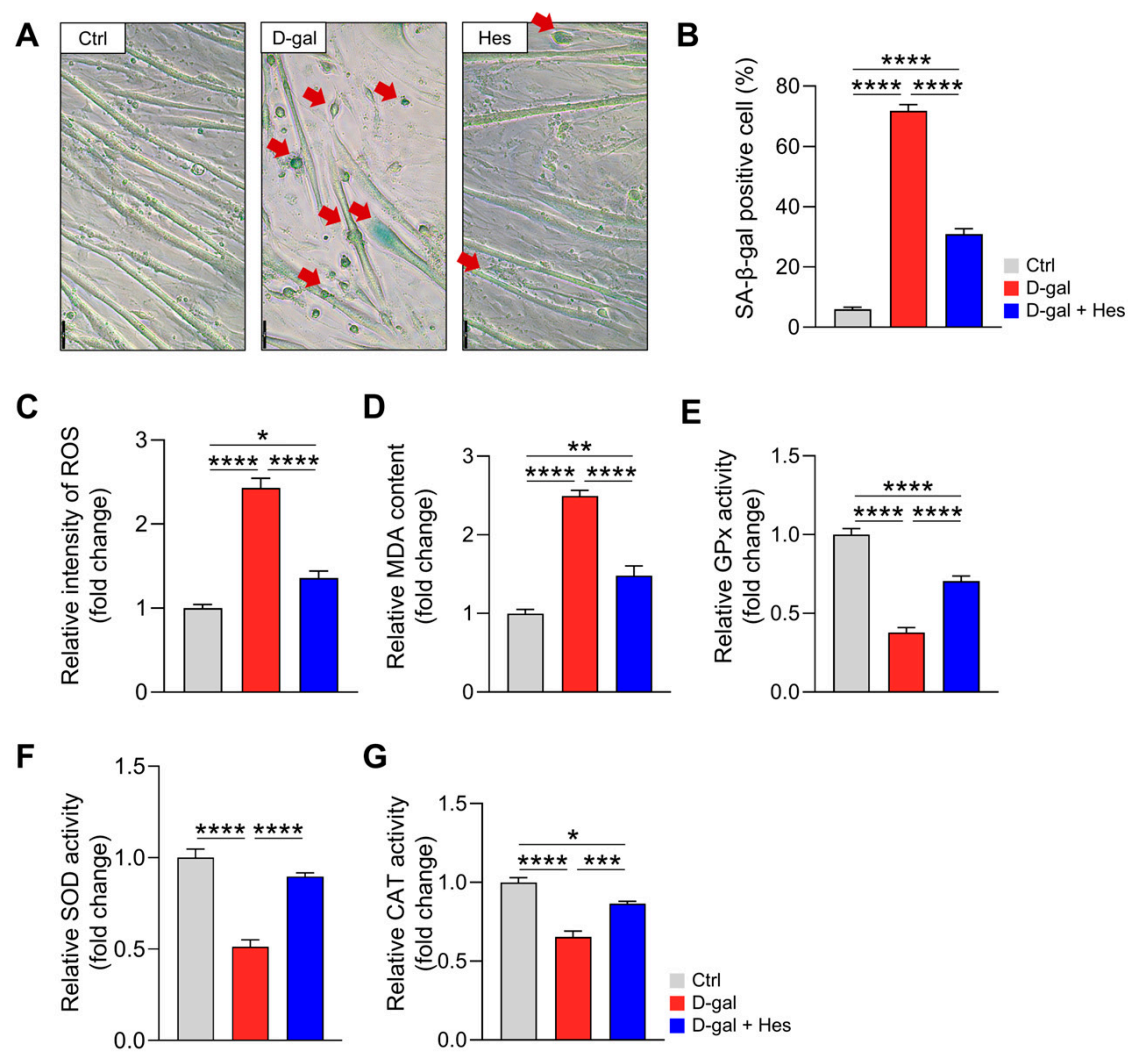
Hesperetin Attenuates D-gal-Induced Cellular Senescence and Oxidative Stress-Related Markers. (**A**) SA-β-galactosidase staining was performed to assess cellular senescence at 5 days after differentiation (red arrows indicate senescent cells). Scale bar = 50 µm. (**B**) Quantification of SA β-gal-positive cells. All the values are presented as mean ± SEM. One-way ANOVA with Tukey’s multiple comparisons. Intracellular ROS levels (**C**) and MDA content (**D**) were also measured (*n* = 6/group). (**E**–**G**) Enzymatic activities of GPx, SOD, and CAT were determined (*n* = 6/group). Antioxidant enzyme activities were normalized to total protein content and expressed relative to the control group. All the values are presented as mean ± SEM. One-way ANOVA with Tukey’s multiple comparisons. * *p* < 0.05, ** *p* < 0.01, *** *p* < 0.001, **** *p* < 0.0001 (One-way ANOVA). Ctrl; Control, D-gal; D-galactose, Hes; Hesperetin, ROS; Reactive oxygen species, MDA; Malondialdehyde, GPx; Glutathione peroxidase, SOD; Superoxide dismutase, CAT; Catalase.

To evaluate the effect of hesperetin on D-gal-induced cellular senescence, SA-β-gal staining was performed in differentiated C2C12 cells (Figure 9A). The percentage of SA-β-gal positive cells markedly increased by 65.9% in the D-gal group compared with the control group and decreased by 40.9% following hesperetin treatment compared with the D-gal group (*p* < 0.0001; Figure 9B). SA-β-gal-staining revealed that hesperetin treatment significantly attenuated D-gal-induced cellular senescence in differentiated C2C12 cells. Furthermore, hesperetin treatment substantially attenuated the expression of oxidative stress markers associated with cellular senescence. Both DCF-DA fluorescence and MDA levels were significantly higher in the D-gal group than in the control group, increasing by 143.2% and 149.3%, respectively (Figure 9 C,D). Hesperetin treatment significantly reduced ROS and MDA levels by 44.1% and 40.6%, respectively, compared with the D-gal group. In contrast, hesperetin treatment markedly reduced the DCF-DA fluorescence and MDA levels, suggesting attenuation of oxidative stress-related markers. In addition, the activities of GPx, SOD, and CAT were significantly decreased by 62.1%, 48.6%, and 34.6%, respectively, in the D-gal group compared to those in the control group (Figure 9E–G). Hesperetin treatment restored these enzymatic activity levels and significantly increased GPx by 85.7% (*p* < 0.0001), SOD by 74.6% (*p* < 0.0001), and CAT by 32.3% (*p* < 0.001) activity. Overall, these findings suggest that hesperetin attenuates D-gal-induced cellular senescence in association with improved redox balance-related markers and antioxidant enzyme activities.

### 3.8. Hesperetin Improves Mitochondrial Function in D-Gal-Induced Senescent C2C12 Cells

**Figure 10:**
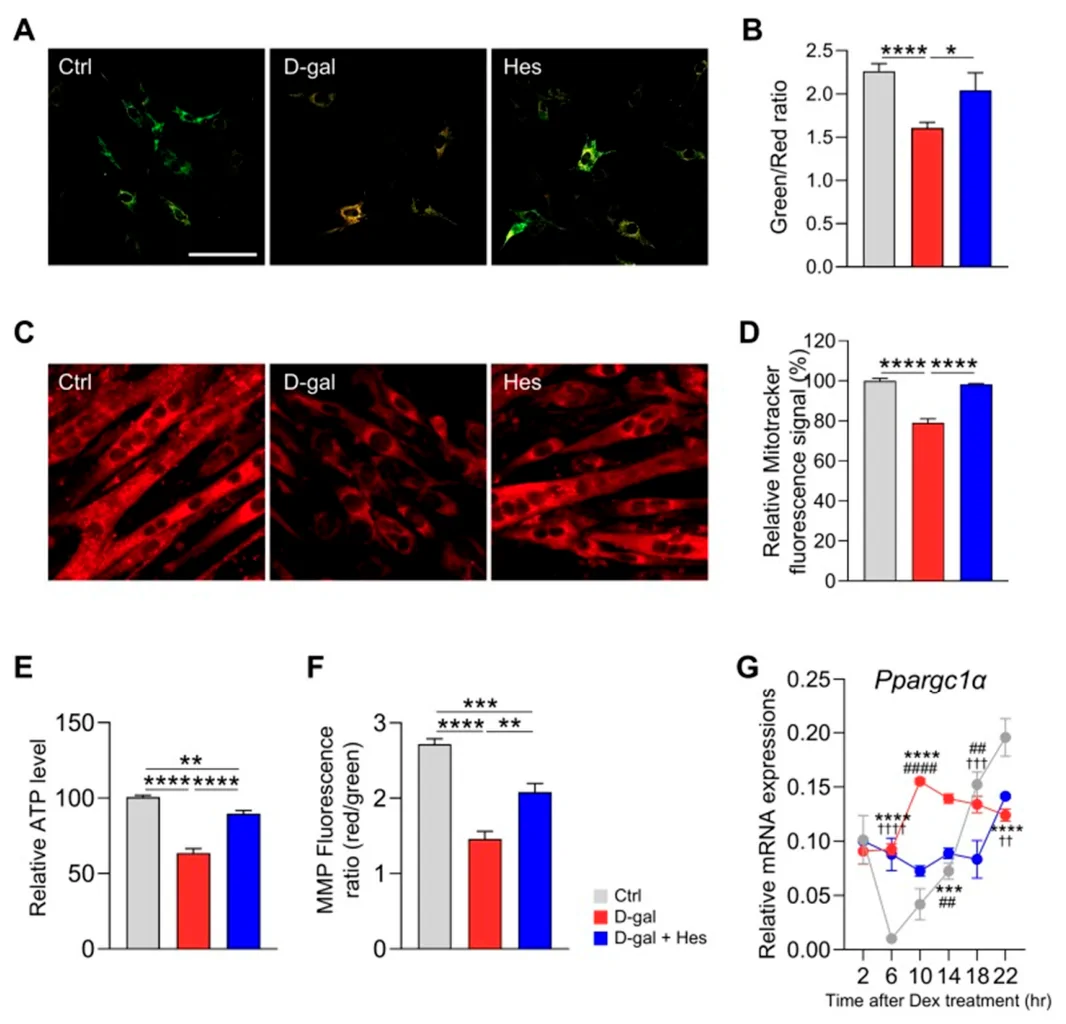
Hesperetin Enhances Mitochondrial-Associated Functional Indicators in D-Gal–Induced Senescent C2C12 Cells. (**A**) Representative images of *pMitoTimer* reporter gene expression obtained by confocal microscopy. 20× magnification. Scale bar = 100 µm. (**B**) Quantification of *pMitotimer* Green/red fluorescence intensity. All values are presented as mean ± SEM. One-way ANOVA with Tukey’s multiple comparisons. (**C**) Representative confocal microscopy images of MitoTracker Red CMXRos fluorescence staining. 40× magnification. (**D**) Quantification of MitoTracker fluorescence intensity. Fluorescence intensity was quantified and expressed relative to the control group. All the values are presented as mean ± SEM. One-way ANOVA with Tukey’s multiple comparisons. (**E**) Intracellular ATP levels. ATP levels were normalized to total protein content and expressed relative to the control group. Mitochondrial membrane potential (MMP) and expressed as the red/green fluorescence ratio (**F**), were determined (*n* = 6 per group). All values are presented as mean ± SEM. One-way ANOVA with Tukey’s multiple comparisons. (**G**) *Ppargc1a* expression in C2C12 cells at day 4 after differentiation was analyzed by real-time qPCR. All the values are presented as mean ± SEM every 4 h over a 24-h period following Dex synchronization (*n* = 3/group/time point). *: Crtl vs. D-gal, #: D-gal vs. Hes, †: Ctrl vs. Hes. Two-way ANOVA with Tukey’s multiple comparisons. * *p* < 0.05, ** *p* < 0.01, *** *p* < 0.001, **** *p* < 0.0001, ## *p* < 0.01, #### *p* < 0.0001, †† *p* < 0.01, ††† *p* < 0.001, †††† *p* < 0.0001. Ctrl; Control, D-gal; D-galactose, Hes; Hesperetin, ATP; adenosine triphosphate, MMP; Mitochondrial Membrane Potential.

*pMitoTimer* imaging was performed to assess mitochondrial oxidation-associated fluorescence changes (Figure 10A). Green fluorescence indicated newly synthesized mitochondria, whereas oxidation induced a shift to red fluorescence. Compared with the control group, the green/red fluorescence ratio was significantly decreased by 29.0% in D-gal-induced senescent C2C12 myoblasts (*p* < 0.0001), suggesting increased mitochondrial oxidative aging (Figure 10B). Conversely, after hesperetin treatment, the green/red ratio was significantly elevated by 27.1%, reflecting reduced mitochondrial oxidation-associated fluorescence signals in D-gal-induced senescent muscle cells (*p* < 0.05). MitoTracker Red CMXRos staining was performed to assess mitochondria-associated fluorescence in differentiated C2C12 myotubes (Figure 10C). The control group displayed well-differentiated myotubes characterized by multinucleated cells and rich mitochondrial signals. The MitoTracker fluorescence intensity was significantly decreased by 21.0% in D-gal-induced senescent C2C12 myotubes compared with the control group (*p* < 0.0001). Hesperetin treatment significantly increased MitoTracker fluorescence intensity by 19.3% compared with the D-gal group (*p* < 0.0001), partially restoring mitochondria-associated fluorescence intensity toward the control level (Figure 10D). Consistent with the changes in MitoTracker fluorescence intensity, intracellular ATP levels were assessed. Compared to the control group, the D-gal group showed an approximately 36.8% decrease in ATP levels (*p* < 0.0001) (Figure 10E). Hesperetin treatment significantly restored ATP levels by 41.1% compared with the D-gal group (*p* < 0.0001). Similarly, Mitochondrial membrane potential (MMP), measured by the red/green fluorescence ratio (Figure 10F), was significantly decreased by 46.2% in the D-gal group compared to the control group (*p* < 0.0001), consistent with impaired mitochondrial membrane potential-related indicators. Hesperetin treatment markedly increased MMP, as indicated by an elevated red/green fluorescence ratio by 42.5% compared with the D-gal group (*p* < 0.01). Peroxisome proliferative activated receptor, gamma, coactivator 1 alpha (*Ppargc1a*) mRNA expression, a key regulator of mitochondrial function, displayed an anti-phase pattern in D-gal-induced senescent C2C12 myotubes relative to control group (Figure 10G). Hesperetin treatment normalized the rhythmic expression pattern of *Ppargc1a*. Collectively, these results suggest that hesperetin improves mitochondrial-associated functional indicators in D-gal-induced senescent C2C12 myotubes, as reflected by increased mitochondria-associated fluorescence, higher ATP levels, improved mitochondrial membrane potential-related fluorescence, reduced *pMitoTimer*-based mitochondrial oxidation-associated signals, and altered *Ppargc1a* expression patterns.

## 4. Discussion

In this study, hesperetin was found to mitigate D-galactose-induced aging-like skeletal muscle dysfunction. This effect was associated with alterations in time-of-day-dependent gene expression, reduction in oxidative stress markers, and enhancements in mitochondrial-related functional indicators in both C2C12 myotubes and D-gal-treated mice. Notably, hesperetin significantly improved performance in the hanging test during the active phase, ZT16, where D-gal reduced hanging time by 57.2% compared with the control group, whereas hesperetin increased hanging time by 100.5% compared with the D-gal group. However, this should be interpreted as an enhancement in overall motor performance rather than direct evidence of increased hanging test performance. Collectively, these findings indicate that hesperetin may offer protection against experimentally induced skeletal muscle dysfunction by modulating redox balance, mitochondrial function, and time-of-day-dependent muscle responses. The D-gal- and Dex-induced models were used to represent distinct but complementary aspects of skeletal muscle deterioration. These models do not fully reproduce the complexity of natural muscle aging or clinical sarcopenia, but they provide useful experimental systems for evaluating muscle dysfunction under different stress conditions. The D-gal model primarily reflects aging-associated functional and mitochondrial impairments, which are commonly linked to oxidative stress, glycation-related damage, and senescence-like changes [34], as shown by disrupted muscle performance and altered diurnal regulation of mitochondrial oxidative capacity without evident changes in body weight or muscle fiber type composition. In contrast, the Dex model represents an atrophy-associated condition characterized by body weight loss and reduced muscle fiber size [35,36,37]. Thus, these two models allowed us to distinguish between mitochondrial functional disruption and structural muscle atrophy, both of which contribute to age-related skeletal muscle decline. This dual-model approach allowed us to evaluate the effects of hesperetin on both metabolic/mitochondrial dysfunction and structural muscle atrophy. For this reason, the effects of hesperetin were interpreted within each experimental model rather than as direct evidence that hesperetin prevents natural muscle aging or sarcopenia. Therefore, these two models allowed us to examine the effects of hesperetin across aging-like metabolic/mitochondrial-associated dysfunction and glucocorticoid-mediated catabolic muscle atrophy, while considering the distinct biological context of each model.

Dex-induced muscle atrophy is characterized by increased muscle protein degradation, accompanied by increased expression of *Fbxo32* and *Trim63*, suppression of Akt/mTOR signaling, and mitochondrial dysfunction [35]. Consistent with these characteristics, Dex treatment reduced muscle fiber size and disrupted the rhythmic expression of *Fbxo32*, *Trim63*, *Pax7*, and *Myod1*. Hesperetin partially restored these changes, suggesting that it attenuates Dex-induced skeletal muscle atrophy by improving both muscle atrophy- and differentiation-related responses.

ZT6 and ZT18 were selected to represent the middle of the light/rest and dark/active phases of the in vivo time-of-day-dependent cycle, respectively. These time points were chosen based on previous studies showing pronounced time-dependent variations at CT6 and CT18 in C2C12 cells and at ZT6 and ZT18 in skeletal muscle tissues [37,38,39]. Aging is commonly associated with reduced diurnal amplitude, altered phase timing, and disrupted rhythmic coordination in the peripheral tissues [40]. Because skeletal muscles possess a robust peripheral clock that regulates energy metabolism, mitochondrial function, contractile capacity, and regenerative processes, disruption of this clock may contribute to impaired muscle homeostasis and age-related muscle degeneration [41,42,43,44,45,46,47,48,49,50,51,52,53]. In this context, our findings suggest that hesperetin improves skeletal muscle aging by restoring the coordination between circadian clock regulation and myogenic differentiation. D-gal-induced senescence disrupted both core clock gene rhythmicity and the expression patterns of myogenic regulatory factors, which is consistent with previous reports linking circadian disruption to impaired myogenesis and reduced regenerative capacity [51,54,55,56]. Hesperetin treatment partially restored these altered rhythmic patterns and improved myotube differentiation, suggesting that recovery of skeletal muscle time-dependent rhythmicity may support the maintenance of myogenic capacity under aging-related stress. Although two time points do not allow complete characterization of time-dependent parameters, the ZT6/ZT18 comparison provides evidence of altered diurnal expression patterns in skeletal muscle. Therefore, partial restoration of diurnal clock gene expression may represent one mechanism by which hesperetin attenuates age-related skeletal muscle degeneration.

Mitochondrial dysfunction and oxidative stress are central mechanisms linking circadian disruption to skeletal muscle aging [32,57,58,59,60,61,62,63,64,65]. During aging, excessive ROS generation can damage mitochondrial DNA, lipids, and proteins, thereby impairing the mitochondrial membrane potential, ATP production, and overall bioenergetic capacity [66,67,68]. Because skeletal muscle has a high energy demand, disruption of mitochondrial redox homeostasis may directly contribute to reduced muscle performance, impaired contractile function, and age-related decline in muscle performance. Therefore, antioxidant defense systems, including SOD, CAT, and GPx, are essential for maintaining the redox balance and protecting skeletal muscles against aging-associated oxidative damage [69,70]. This mitochondrial-centered interpretation is consistent with the absence of marked body weight loss or fiber-type changes in the D-gal model.

The recovery of antioxidant enzyme activity, mitochondria-associated fluorescence, ATP levels, and mitochondrial membrane potential-related fluorescence suggests that hesperetin may support skeletal muscle function by modulating mitochondrial-associated bioenergetic and redox-related indicators. In D-gal-induced senescent C2C12 cells, D-gal decreased ATP levels by 36.8% and mitochondrial membrane potential-related fluorescence by 46.2%, whereas hesperetin increased these parameters by 41.1% and 42.5%, respectively, compared with the D-gal group. In addition, hesperetin improved the altered expression pattern of *Ppargc1a*, a central regulator of mitochondrial biogenesis and a circadian-regulated gene, supporting the possibility that the mitochondrial protective effects of hesperetin are linked to time-of-day-dependent regulation of mitochondrial metabolism [71,72,73,74]. Hesperetin also altered the expression patterns of *Ucp2* and *Ucp3*, which are involved in mitochondrial redox regulation and uncoupling. However, because UCP2 and UCP3 can influence mitochondrial membrane potential and ATP synthesis, these changes should be interpreted as mitochondrial regulatory responses rather than as uniformly beneficial indicators of improved mitochondrial function [75,76]. Consistent with this interpretation, hesperetin increased SDH-positive area and improved hanging test performance more prominently during the active phase without altering muscle fiber composition. In the D-gal experiment, D-gal reduced SDH-positive area by 51.0% at ZT6 and 82.5% at ZT18, while hesperetin significantly attenuated this reduction. These findings suggest that hesperetin may influence metabolic and mitochondrial-associated components of skeletal muscle function rather than inducing major structural changes in fiber-type distribution.

Hesperetin treatment attenuated D-gal-induced oxidative stress-related markers and improved mitochondrial-associated functional indicators in both cellular and animal models. In particular, in D-gal-induced senescent C2C12 cells, D-gal decreased GPx, SOD, and CAT activities by 62.1%, 48.6%, and 34.6%, respectively, compared with the control group, whereas hesperetin increased these enzyme activities by 85.7%, 74.6%, and 32.3%, respectively, compared with the D-gal group. These findings are in line with previous evidence supporting the antioxidant and mitochondrial effects of hesperetin. For example, Shen et al. reported that hesperetin enhanced mitochondrial respiration, reduced ROS accumulation in aged human keratinocytes, and attenuated age-related skin deterioration in naturally aged mice through CISD2 activation [77]. However, the mitochondrial- and redox-related findings in the present study should be interpreted with caution. Although ATP levels and antioxidant enzyme activities were normalized to total protein content, the observed changes may still be influenced by differences in cell viability, myotube differentiation, or overall cellular status. Therefore, these findings should be interpreted as changes in mitochondrial-associated and redox-related indicators rather than direct evidence of mitochondrial respiratory mechanism or specific antioxidant pathway. Furthermore, using the *pMitoTimer* reporter system, we observed that hesperetin attenuated mitochondrial oxidation-associated fluorescence signals in senescent muscle cells. These results suggest that time-of-day-dependent gene expression and mitochondrial redox-related regulation may contribute to the protective effects of hesperetin.

More specifically, previous skeletal muscle studies have shown that hesperetin improves mitochondrial bioenergetics in myotubes, attenuates oxidative stress-related responses in skeletal muscle cell models, and enhances myogenic differentiation under oxidative stress conditions [25,26,78]. The present study extends these findings by combining time-resolved in vitro analyses with ZT-based in vivo sampling and by integrating multiple features of aging-like and catabolic skeletal muscle dysfunction, including redox imbalance, mitochondrial-associated functional impairment, impaired myogenic responses, and muscle atrophy. Notably, the improvement in muscle performance, as reflected by the prolonged hanging time, was more evident during the active phase (ZT16–ZT18), despite no significant changes in muscle fiber composition. This suggests that the functional improvement induced by hesperetin in the D-gal-induced aging model may be primarily associated with metabolic and mitochondrial adaptations rather than with fiber-type remodeling. This interpretation is supported by recent studies showing that skeletal muscle *Bmal1* disruption or restoration can markedly alter transcriptomic, metabolic, and functional profiles without necessarily changing the muscle fiber size or fiber-type composition [79,80]. Together, these findings suggest that hesperetin may act across distinct but complementary aspects of skeletal muscle dysfunction, while the pattern of its effects appears to depend on both the underlying experimental condition and the time of assessment.

Comparison of the two experimental models revealed both shared and model-dependent skeletal muscle responses to hesperetin. The D-gal model showed aging-like changes associated with altered myogenic markers, time-of-day-dependent clock gene expression, mitochondrial-associated indicators [33], and oxidative stress-related responses. However, Dex produced a more profound catabolic phenotype with smaller muscle fiber size and altered expression of atrophy-, myogenesis-, and metabolism-related genes [34]. In the Dex model, final body weight was reduced by 11.3%, and mean muscle fiber CSA was reduced by 42.0%, 37.0%, and 23.1% in the gastrocnemius, plantaris, and soleus muscles, respectively. Hesperetin increased body weight by 9.8% and increased mean CSA by 15.0%, 20.1%, and 17.9% in these muscles, respectively, compared with the Dex group. These results are in line with the well-reported features of glucocorticoid-induced muscle atrophy [34,35,36], where Dex leads to activation of proteolytic and catabolic responses rather than mark senescence-like oxidative/glycation stress [35] in which Dex promotes proteolytic and catabolic responses rather than primarily inducing senescence-like oxidative/glycation stress [34]. In the Dex model, hesperetin partially attenuated the reduction in muscle fiber size and altered the expression of selected genes involved in the muscle atrophy and myogenesis, indicating protective effect against glucocorticoid-induced muscle catabolism. Differences between two models likely reflect their distinct biological characteristics: D-gal induces senescence-like changes largely associated with oxidative and glycation stress, whereas dex promotes muscle catabolism through glucocorticoid signaling. Notably, the SDH-positive area exhibited different patterns across the two experiments, even in the respective control groups. SDH-related outcomes were therefore evaluated relative to the control group within each model, rather than compared quantitatively between models. Although hesperetin improved selected muscle, mitochondrial, and redox outcomes in both models, the magnitude and pattern of these differed responses. Together, these findings support protective role for hesperetin under distinct forms of experimentally induced muscle dysfunction. Nevertheless, because the two models involve different pathological processes and were examined in separate experiments, the findings do not establish a common underlying mechanism of hesperetin action.

Despite these promising findings, this study had several limitations. First, although hesperetin partially restored the circadian gene oscillations, the detailed molecular mechanisms underlying this effect remain unclear. Further studies are required to determine whether hesperetin directly modulates core clock transcriptional regulators or indirectly affects circadian rhythmicity through mitochondrial function and redox homeostasis. Second, although mitochondrial function and oxidative stress were examined, this study did not directly assess upstream signaling pathways linking hesperetin to clock regulation, mitochondrial biogenesis, or proteolytic systems such as ubiquitin–proteasome and autophagy–lysosome pathways. Future studies should investigate whether hesperetin regulates these pathways and contributes to the protection against skeletal muscle aging. Third, gene expression in the in vivo experiments was evaluated at only two time points, ZT6 and ZT18. These data may suggest differences at a specific selected time of day, but cannot provide information on circadian rhythmicity phase, amplitude, or period. Future experiments over the entirety of a 24 h period with rhythmicity analysis are necessary to estimate the circadian effects of hesperetin in skeletal muscle. Fourth, this study included only male mice which may affect the generalizability of the results. Lastly, since circadian rhythms, redox control of mitochondrial metabolism, and skeletal muscle metabolism are controlled by complex interorgan cooperativity, additional in vivo experiments are needed to elucidate the system-wide effects of hesperetin during aging-like and catabolic stress conditions. Also, pharmacokinetic experiments measuring tissue drug concentrations, circulating bioavailability, and hesperetin metabolites are needed to better translate the doses utilized in this study.

Taken together, our findings indicate that hesperetin attenuates selected features of skeletal muscle dysfunction in experimentally induced aging-like and glucocorticoid-mediated catabolic models. These effects were accompanied by changes in time-of-day-dependent gene expression and mitochondrial and redox-related outcomes, supporting the potential involvement of temporal and metabolic regulation in the protective actions of hesperetin. Nevertheless, comprehensive circadian profiling, direct measurements of mitochondrial respiration, dose–response studies, and further mechanistic experiments are required to define how hesperetin influences skeletal muscle homeostasis under these distinct stress conditions.

## 5. Conclusions

This study shows that hesperetin attenuated selected features of experimentally induced skeletal muscle dysfunction, accompanied by changes in time-of-day-dependent gene expression, oxidative stress markers, and mitochondrial-related functional measures. In D-gal-treated myotubes and mice, hesperetin improved markers of myogenic differentiation, redox homeostasis, and mitochondrial function, along with hanging test performance. In the Dex-induced catabolic atrophy model, hesperetin also attenuated the reduction in muscle fiber size, with the evident effect observed in the gastrocnemius muscle. Together, these findings provide basis for further investigation of hesperetin as a dietary bioactive compound that may help preserve skeletal muscle homeostasis under aging-like and catabolic stress conditions.

## Figures and Tables

**Table 1:** Primer sequences for RT-qPCR.

Gene Symbol	Accession Numbers	Forward (5′-3′)	Reverse (5′-3′)
*Gapdh*	NM_008084.4	CAAGGTCATCCATGACAACTTTG	GGCCATCCACAGTCTTCTGG
*Bmal1*	NM_007489.5	CCACCTCAGAGCCATTGATACA	GAGCAGGTTTAGTTCCACTTTGTC
*Per* *1*	NM_011065.5	TTCGTGGACTTGACACCTCTT	GGGAACGCTTTGCTTTAGAT
*Per2*	NM_011066.4	ATGCTCGCCATCCACAAGA	GCGGAATCGAATGGGAGAAT
*Cry1*	NM_007771.4	CTGGCGTGGAAGTCATCGT	CTGTCCGCCATTGAGTTCTATG
*Cry* *2*	NM_009963.4	TGTCCCTTCCTGTGTGGAAGA	GCTCCCAGCTTGGCTTGA
*Dbp*	NM_016974.4	CTGGCCCGAGTCTTTTTGC	CCAGGTCCACGTATTCCACG
*Nr1d1*	NM_145434.4	CATGGTGCTACTGTGTAAGGTGT	CACAGGCGTGCACTCCATAG
*Rora*	NM_013646.3	GCACCTGACCGAAGACGAAA	GAGCGATCCGCTGACATCA
*Ror* *c*	NM_011281.4	TCAGCGCCCTGTGTTTTTC	GAGAACCAGGGCCGTGTAG
*Ucp2*	NM_011671.6	ATGGTTGGTTTCAAGGCCACA	CGGTATCCAGAGGGAAAGTGAT
*Ucp* *3*	NM_009464.3	TGGCCCAACATCACAAGAAA	TCCAGCAACTTCTCCTTGATGA
*Myf5*	NM_008656.5	CTGTCTGGTCCCAAAGAAC	TGGAGAGAGGGAAGCTGTGT
*Myod1*	NM_010866.2	GCACTACAGTGGCGACTCAGAT	TAGTAGGCGGTGTCGTAGCCAT
*Myog*	NM_031189.2	AGTGAATGCAACTCCCACAG	ACGATGGACGTAAGGGAGTG
*Myf6*	NM_008657.3	GGGCCTCGTGATAACTGCTA	CCTGCTGGGTGAAGAATGTT
*Ppargc1a*	NM_008904.3	AACCACACCCACAGGATCAGA	CTCTTCGCTTTATTGCTCCATG
*Fbxo32*	NM_026346.3	CATCCCTGAGTGGCATCG	GAGTCTGGAGAAGTTCCCGTAT
*Trim63*	NM_001039048.2	TACCAAGCCTGTGGTCATCCTG	ACGGAAACGACCTCCAGACATG
*Pax7*	NM_011039.3	CTGCTCTGAGCCCACCAG	GACAGGGCTGTTACATTCAGG

## Data Availability

All of the data is contained within the article and the Supplementary Materials.

## Supplementary Materials

The following supporting information can be downloaded at: https://www.mdpi.com/article/10.3390/antiox15091185/s1. Figure S1. Hesperetin Attenuates on Skeletal Muscle Atrophy in a Dex–Induced Atrophy Mouse Model. Figure S2. Diurnal expression of muscle atrophy and differentiation genes in a Dex–Induced Atrophy Mouse Model.
