# Supplementary material for: Hesperetin Attenuates Experimentally Induced Skeletal Muscle Dysfunction by Modulating Time-of-Day-Dependent Gene Expression and Mitochondrial Redox-Related Markers
Source: Antioxidants (Basel). 2026 Sep 17;15(9):1185. doi: 10.3390/antiox15091185 (PMC13603285; doi:10.3390/antiox15091185)

Supplementary Figure S2.

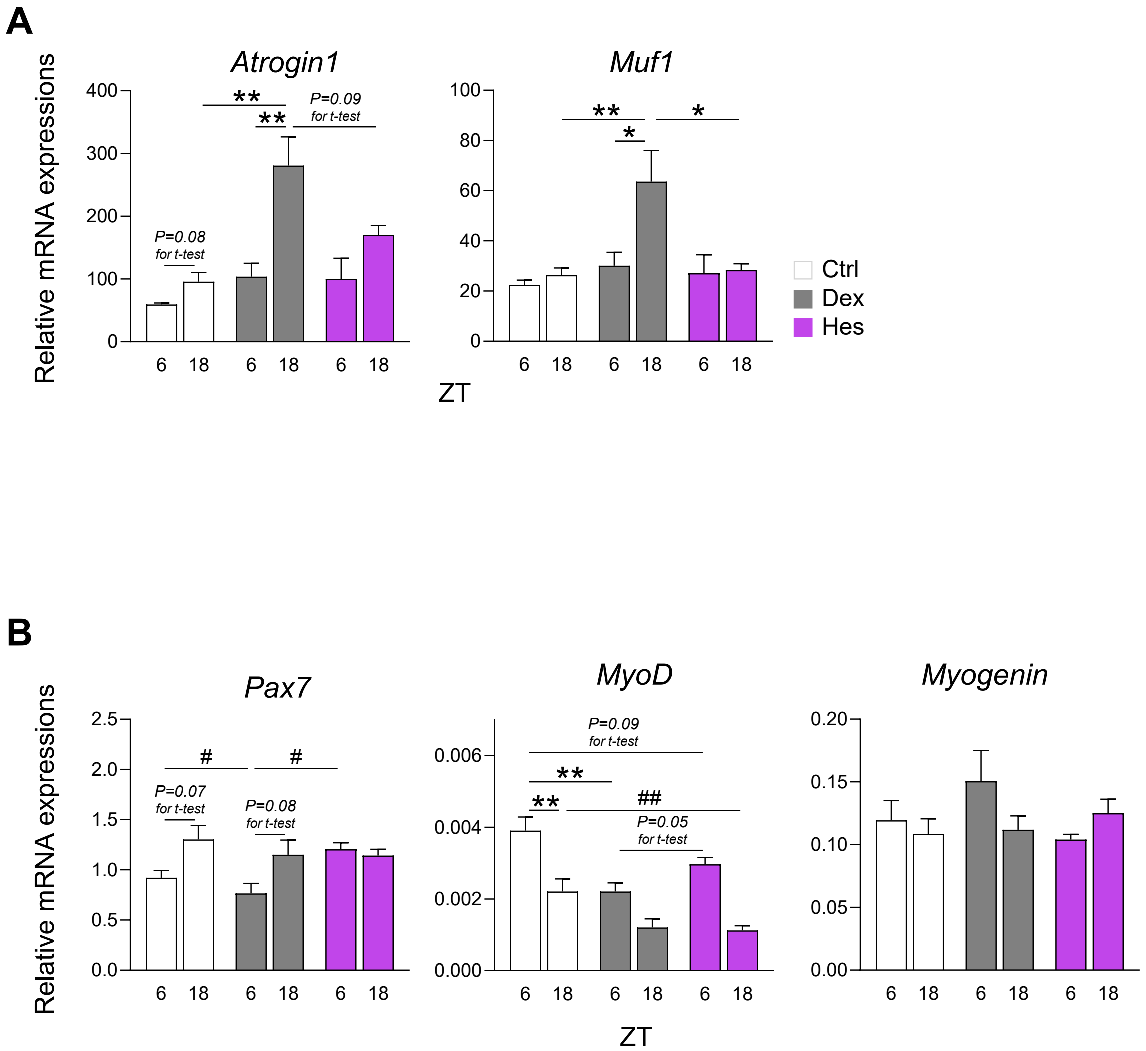

Supplement: Supplementary file 1 [file antioxidants-15-01185-s001.zip › Supplementary Figure S2.pdf]
