# Supplementary material for: Hesperetin Attenuates Experimentally Induced Skeletal Muscle Dysfunction by Modulating Time-of-Day-Dependent Gene Expression and Mitochondrial Redox-Related Markers
Source: Antioxidants (Basel). 2026 Sep 17;15(9):1185. doi: 10.3390/antiox15091185 (PMC13603285; doi:10.3390/antiox15091185)
Supplement: Supplementary file 1 [file antioxidants-15-01185-s001.zip › Supplementary Figures.pdf]

Supplementary Figures

Supplementary Figure S1. Hesperetin Attenuates on Skeletal Muscle Atrophy in a Dex–Induced Atrophy Mouse Model.

A. Experimental overview. **B.** Mouse body weight analysis (n = 8/group). All the values are presented as mean ± SEM. One-way ANOVA with Tukey's multiple comparisons. **C.** Quantification of the muscle fiber area in calf tissues by SDH staining image. CSA; cross-sectional area. **D**. Confocal microscopy analysis of muscle fiber type in soleus and plantaris muscle (Blue: Type I, green: Type IIA, red: Type IIB). 40X magnification. Scale bar: 50 μm. **E.** Succinate dehydrogenase (SDH) staining in soleus muscle. 20X magnification. Scale bar: 100 μm. **F.** Quantification of the proportion of SDH positive fibers in soleus muscle at ZT6 and ZT18 (Threshold: 170). Data are shown as mean ± SEM. Two-way ANOVA with Tukey's multiple comparisons. * $p < 0.05$, ** $p < 0.01$, *** $p < 0.001$, **** $p < 0.0001$ (Two-way ANOVA). ## $p < 0.01$ (Welch's t-test). Dex; Dexamethasone, Hes; Hesperetin, ZT; Zeitgeber Time.

Supplementary Figure S2. Diurnal expression of muscle atrophy and differentiation genes in a Dex–Induced Atrophy Mouse Model.

A-B. Expression of genes related to muscle atrophy (A) and muscle differentiation (B) in calf tissues at ZT6 and ZT18 was analyzed by real-time qPCR. All the values are presented as mean ± SEM (n=3-4/group/time point). Two-way ANOVA with Tukey's multiple comparisons and Welch's t-test. * $p < 0.05$, ** $p < 0.01$ (Two-way ANOVA). # $p < 0.05$, ## $p < 0.01$ (Welch's t-test). Dex; Dexamethasone, Hes; Hesperetin, ZT; Zeitgeber Time.
